# Recent Advances on Early-Stage Fire-Warning Systems: Mechanism, Performance, and Perspective

**DOI:** 10.1007/s40820-022-00938-x

**Published:** 2022-10-06

**Authors:** Xiaolu Li, Antonio Vázquez-López, José Sánchez del Río Sáez, De-Yi Wang

**Affiliations:** 1 IMDEA Materials Institute, C/Eric Kandel, 2, 28906 Getafe, Madrid, Spain; 2 E.T.S. de Ingenieros de Caminos, Universidad Politécnica de Madrid, Calle Profesor Aranguren 3, 28040 Madrid, Spain; 3 Departamento de Ingeniería Eléctrica, Electrónica Automática y Física Aplicada, ETSIDI, Universidad Politécnica de Madrid, Ronda de Valencia 3, 28012 Madrid, Spain

**Keywords:** Smart thermosensitive fire sensors, Working mechanism, Response time, Signal conversion

## Abstract

Early-stage fire-warning systems (EFWSs) have attracted significant attention owing to their superiority in detecting fire situations occurring in the pre-combustion process. Substantial progress on EFWSs has been achieved recently, and they have presented a considerable possibility for more evacuation time to control constant unintentional fire hazards in our daily life. This review mainly makes a comprehensive summary of the current EFWSs, including the working mechanisms and their performance. According to the different working mechanisms, fire alarms can be classified into graphene oxide-based fire alarms, semiconductor-based fire alarms, thermoelectric-based fire alarms, and fire alarms on other working mechanisms. Finally, the challenge and prospect for EFWSs are briefly provided by comparing the art of state of fire alarms. This work can propose a more comprehensive understanding of EFWSs and a guideline for the cutting-edge development direction of EFWSs for readers.

## Highlights

- Thermosensitive fire alarms with various working mechanisms are overviewed.
- Different calculation methods for response time are discussed.
- Warning signal conversion types are provided.
- Limitations, challenges, and development direction are put forward.

## Graphical abstract

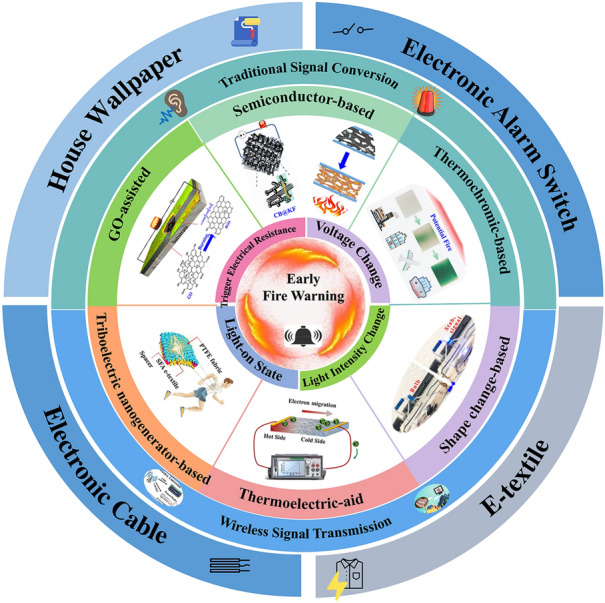

## Introduction

Fire, a kind of vital energy form to sustain the global ecosystem, can also become a global hazard, leading to a catastrophic impact on the world including nature, animals, and humans, as well as serious economic expense [1–4]. Fire hazards can happen in all aspects of society, from devastating wildfires to various indoor fire disasters. The high temperature and smoke and toxic gases during the combustion process will cause destructive harm to the human body [4–7]. Many activities about fire prevention have been carried out to mitigate or avoid its negative influence [8–17].

**Fig. 1:**
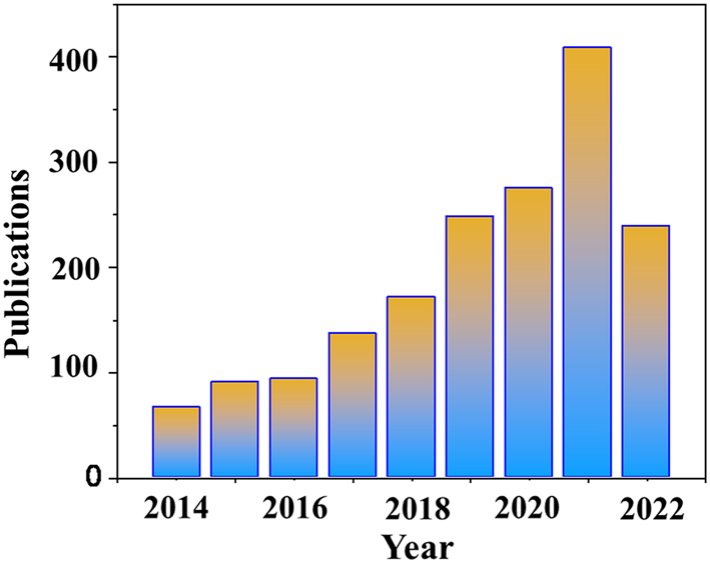
The statistics of publication on EFWSs in recent years

As it is well known, combustion behavior closely involves the ignition temperatures, oxidant, oxygen content (generally the oxygen from the air), and flammable materials [18, 19]. After encountering a heat source, the thermic pyrolysis reaction and decomposition take place, causing a localized temperature increase and a large amount of volatile compounds [20–23]. Afterward, a violent burning reaction would happen to form a fire hazard. To make more efficient fire management, fire sensors have become a desirable strategy. One traditional fire alarm is gas sensors to detect the gas mixture by certain materials which are sensitive to the presence of gaseous compounds [24, 25]. Fire detection using gas sensing has been recognized as a promising approach since the 1990s [26], as different materials can detect the common products from a fire, such as smoke or CO and CO_2_. While this is a successful approach to detecting a fire, predicting a fire is the foremost approach to follow rather than detecting the compounds produced post-combustion. Except for gas detectors, there still are traditional fire-warning systems, consisting of smoke and infrared sensors, for commercial applications [26, 27]. They have the following characteristics: long-distance installation, delayed warning [28], long response time [29], warning after flame triggered, and restricted application under complicated environments. In general, these commercial fire sensors present passable detection capability, but there is still a certain gap in satisfactory sensitivity and applicability. Consequently, sensors capable of detecting fire in the early stage, especially the changes based on the early temperatures developing, are gaining increasing attention, which can be referred to as thermosensitive fire sensors. Considering the fast fire propagation within an extremely short time, developing early smart fire sensors working in the pre-combustion stage is a potential strategy. Certainly, there are many publications on the research of fire sensors in recent years, and the brief statistics are shown in Fig. 1. Publication number has increased rapidly, particularly in these two years. This phenomenon undoubtedly proves the popularity of fire sensors. Specifically, the development of advanced EFWSs has gained considerable attention. Many EFWSs studies have been conducted to accomplish fire monitor. Namely, the subsets of fire sensors are basically referred to as thermosensitive sensors that this review essentially focused on.

Early fire alarms monitor some features displayed in early combustion behavior for warning, so that more time and proper measures can be taken according to the actual situation to avert or minimize losses. In terms of the whole fire-developed process that is composed of incipient, growth, fully developed, decay, and burned-out stages [30], early fire alarms mainly play a positive role in the incipient stage and growth stage. Traditional fire management including additive flame retardant (inhibition of flame spread or self-extinguishing) and firefight methodologies (sprinkler systems [31], water spray systems [32, 33], or water mist [34]), takes effect primarily after extensively fire propagation, implying the “early” in “early fire alarms.” On the other hand, fire will spread into a large area once a violent reaction occurs during combustion. Few materials can withstand the prolonged attack of a fierce fire, which increases the devastation produced by the fire. Fire control is exactly a battle against time. If an abnormal situation can be captured before widespread robust combustion, instant fire management can be enforced to avoid large-scale hazards as much as possible, minimizing the risks and reducing the negative outcomes, which is exactly the advantage offered by early fire alarms. This strong point can make up for the evacuation time delay compared with traditional fire managements.

Two core points of EFWSs contain detecting abnormal behavior and the warning system. Different materials can be selected, and reasonable early warning methods can be employed. From a materials’ point of view, temperature-sensitive materials whose relevant chemical or physical properties are sensitive to thermal energy are commonly adopted to prepare thermosensitive sensors. Thermal sensing materials, comprising semiconductors [35–40], nano-hybrids [41], carbon-based nanomaterials [42–44], conjugated polymers [44], sulfides [45, 46], etc., are acceptable as excellent materials for EFWSs. In these thermosensitive materials, the carbon-based two-dimensional nanomaterial graphene oxide (GO) is a typical representative of fire alarming because of the positive feedback of electrical conductivity to temperatures [47–50]. Moreover, the unique structural character endows GO with fantastic surface properties, thereby leading to superior mechanical strength, high charge carrier mobility, and various sample morphologies [47, 51–53], which can further make GO become the star material for EFWSs. Many early fire-warning studies based on GO have been reported. Likewise, semiconductor materials that possess the special reversible thermally induced conductivity capability can implement the insulator-to-conductor conversion, owning to the charge carrier jump under high-temperature conditions [38, 54]. This performance of semiconductor materials exploits an advantage in distinctive recyclable early warning systems. Moreover, some studies on the basis of sulfides and conjugated polymers have been reported. Recently, thermoelectric (TE) materials [55] and triboelectric nano-generators (TENGs) materials [54] are gradually gaining increasing interest, due to their ability to create electrical signals under different conditions, such as the presence of a temperature. The second core is the warning transmission section. How to quickly transmit detected signals is an important part of EFWSs. The developed transmission system transfers the signal to different devices, which can be simply alert such as lamps, buzzer, or digital device monitors, as well as advanced wireless signal conversion based on the Internet-of-things (loT).

**Scheme 1:**
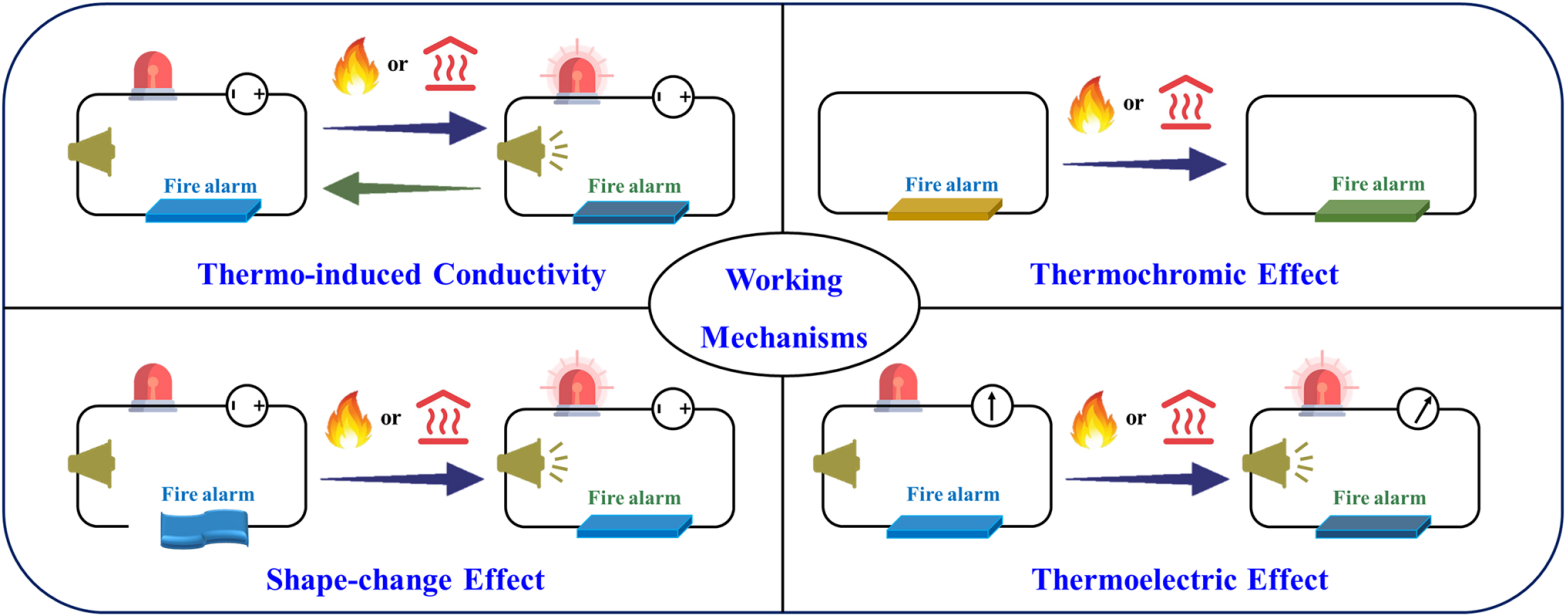
Schematic illustration of the different working mechanisms of EFWSs

The conventional working mechanism for EFWSs is diverse, corresponding schematic description depicted in Scheme 1. In the electrical circuit, including the power supply, cables, warning lamp, and fire-warning samples, the electrical conductivity of warning samples changes under different temperature conditions. One is the transformation from insulation at room temperature to a conductive state after a high temperature or flame attack, which can trigger the warning lamp. The other one is the conductive-to-insulate change upon high temperature or flame attack, leading to the disconnection of an electrical circuit. This is a simple and highly operational working principle to prepare early fire sensors, which has inspired to develop various fire alarming systems. Moreover, thermally induced optical changes are also applied for EFWSs. The color, transmittance, shape, and other characteristics can display difference when warning materials are attacked by high temperature or flame. These particular phenomena can be used as a warning signal. Besides the above-mentioned working principles, other working mechanisms need to be developed to facilitate EFWSs used in different complicated working scenarios. This is a huge challenge for EFWSs development.

**Scheme 2:**
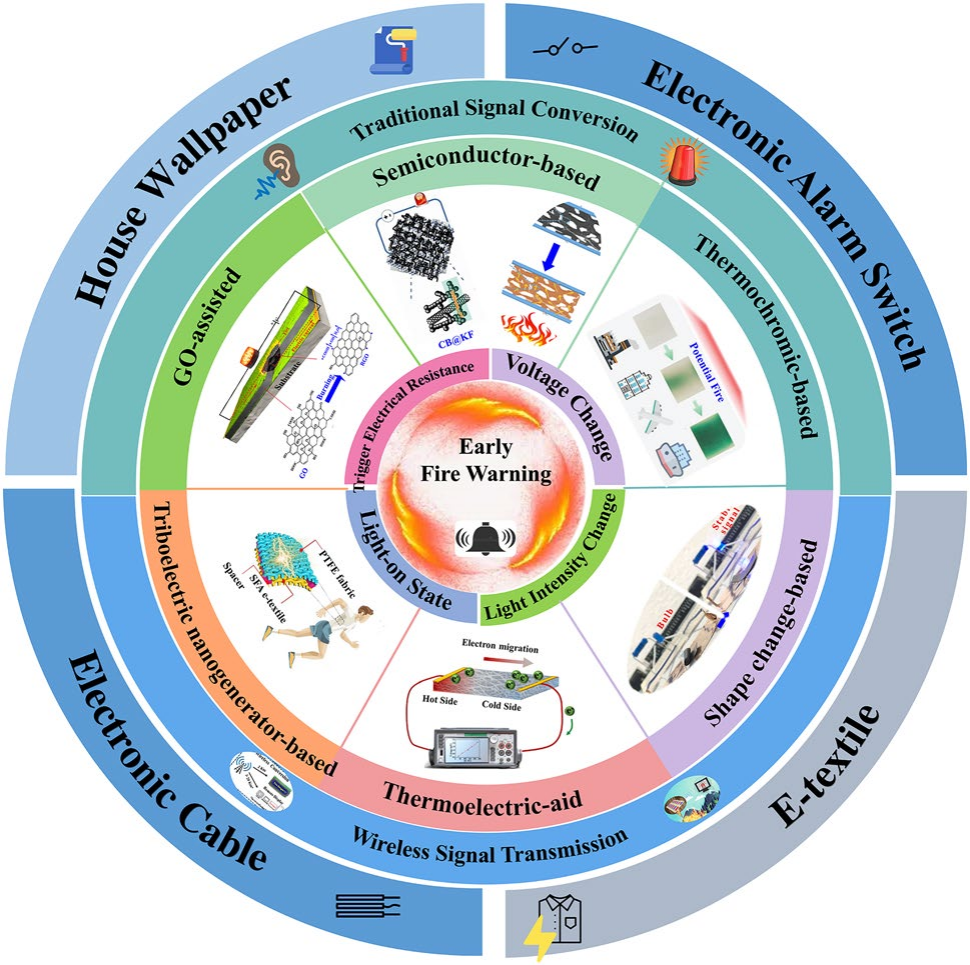
Summary of the issues covered in this review regarding smart EFWSs. GO-assisted fire alarms: reproduced with permission from Ref. [56]. Copyright 2020 Elsevier. Semiconductor-based fire alarms: reproduced with permission from Ref. [43]. Copyright 2022 Elsevier. Schematic preparation of carbon nanotube (CNT) reproduced with permission from Ref. [42]. Copyright 2020 Elsevier. Thermochromic-based fire alarms: reproduced with permission from Ref. [57]. Copyright 2019 Wiley. Shape change-based fire alarms: reproduced with permission from Ref. [58]. Copyright 2021 Elsevier. Thermoelectric-aid fire alarms: reproduced with permission from Ref. [35]. Copyright 2022 Elsevier. Triboelectric-based fire alarms: REPRODUCED with permission from Ref. [54]. Copyright 2022 American Chemistry Society

Herein, the focus of this review is mainly settled on the state-of-the-art thermosensitive EFWSs, which is systematically summarized in Scheme 2, from working mechanisms, response time, and signal conversion to feasible application. These detailed descriptions and discussions based on the above contents are offered in different sections. Furthermore, a summary and comparison of the ever-reported typical EFWSs are depicted in the table, for a comprehensive understanding. Finally, a brief illustration of the current existing challenges and potential developed direction of EFWSs are provided.

## Response Time for EFWSs

The signal detection capability is crucial for EFWSs. In a real fire scenario, a faster detection rate leads to more evacuation time for evacuation, tackling, and controlling the fire. One reply of sensitivity for EFWSs is response time. Based on the current calculation methods of response time, there are four primary standards to count the response time to exhibit the efficiency of early fire alarms. The detailed descriptions are in the following subsections.

### Response Time Based on “Trigger Electrical Resistance”

The conversion of conductive state for electrical pathway under different conditions as the primary working mechanism has been performed well in mostly early fire alarms. This will carry the changes in relevant electrical parameters, such as voltage, resistance, and current. When the warning materials are subjected to high temperature or flame treatment, electrochemical parameters will cause constant changes. During the whole changing process of electrical parameters, there must be one threshold that can change the conductive state to trigger the warning in the electrical circuit. This threshold is the key to judge whether the fire warning can work typically. The time from the beginning until the triggered response can be preliminarily identified as the response time for EFWSs. An electrical resistance threshold is commonly chosen to calculate response time for EFWSs. This electrical resistance threshold is called the “trigger electrical resistance.”

**Fig. 2:**
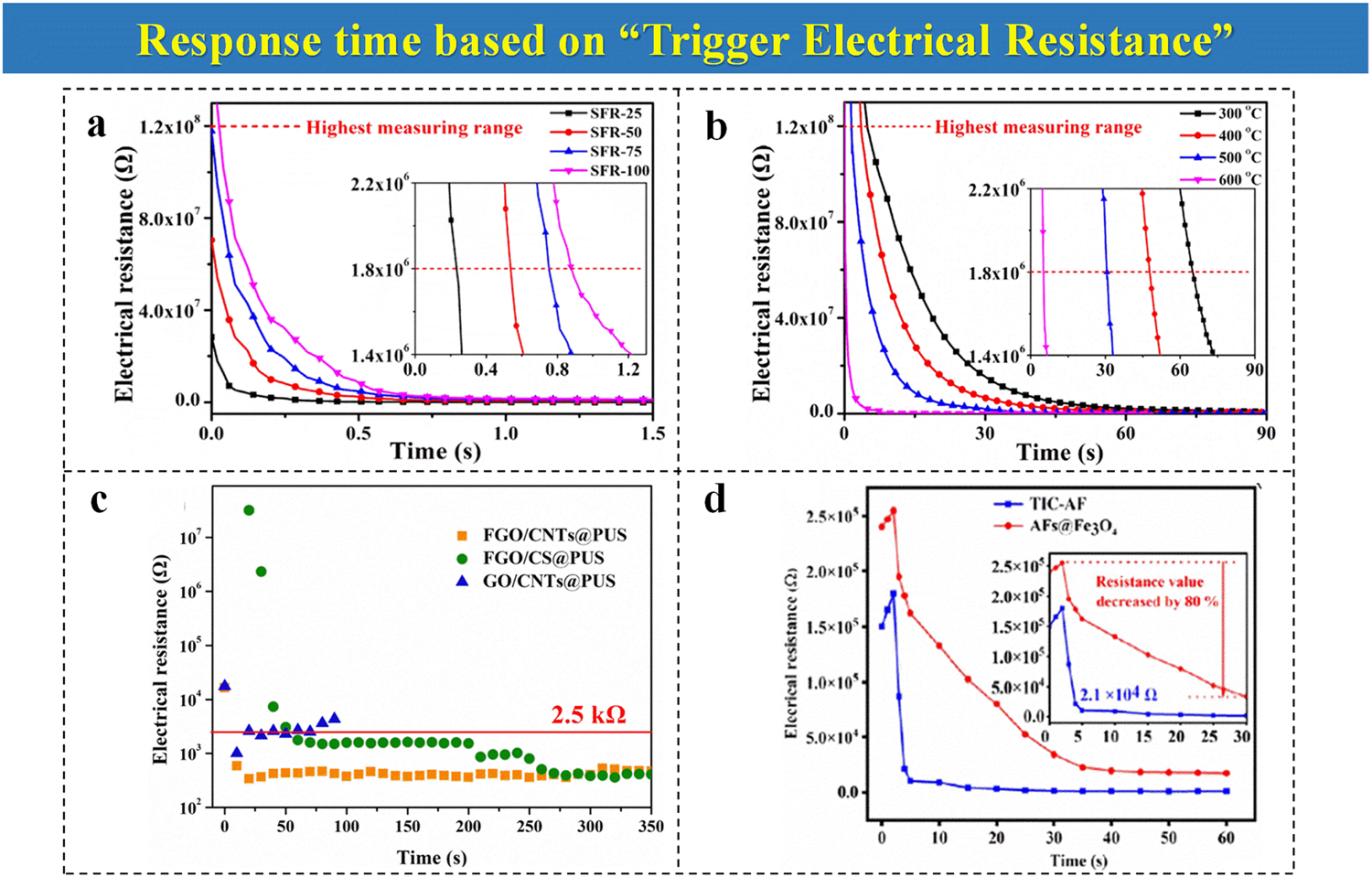
A sandwich-like EFWS: **a** temperature-responsive resistance change curves of different sandwich-like flame-retardant (SFR) nano-coating during the whole fire-warning process. **b** Temperature-responsive resistance curves of SFR-100 nanosheets at different test temperatures treatment. Reproduced with permission from Ref. [56]. Copyright 2020 Elsevier. GO-based PU foam fire-warning system: **c** electrical resistance curves of modified PU foam. Reproduced with permission from Ref. [59]. Copyright 2021 Elsevier. **d** Ultralight fire alarm e-textile system: electrical resistance curves of AF@Fe_3_O_4_ and thermal-induced conductive aerogel fiber (TIC-AFs) during fire-warning process. Reproduced with permission from Ref. [54]. Copyright 2022 American Chemistry Society

This response time calculation method is appropriate for fire-warning systems that can achieve electrical resistance changes. The conventional suitable one is GO-based fire alarming systems, which account for the main proportion of current EFWSs. When GO in the system encounters a high temperature or flame, it suffers thermal reduction reactions, leading to a fast electrical resistance drop to the resistance threshold. As observed in Fig. 2a, b, the initial electrical resistance of warning samples in a sandwich-like early fire alarm is high [56]. After encountering a high temperature, the electrical resistance decreases rapidly within a short test time. To avoid the fluctuations during sample preparation and testing, as well as to maintain the sensitivity and stability of EFWSs, the resistance value that decreases more than 90% compared with initial resistance is set as “trigger electrical resistance” for response time. In fact, the trigger electrical resistance is 1.8 × 10^6^ Ω, 10% of the initial electrical resistance [56]. This value is low enough to ensure the occurrence of the trigger warning, indicating the sensitivity of EFWSs accurately. Moreover, a similar triggering method has been employed in the multi-functional GO-modified polyurethane (PU) foam system (Fig. 2c). In this study, the value of 2.5 × 10^3^ Ω is applied as trigger electrical resistance to get an alarming time, according to their previous experience [59]. This response time is a judgment basis to compare the sensitivity of different fire-warning samples. Likewise, the same approach to calculating response time is consistent in the fire-warning e-textile system [54]. As depicted in Fig. 2d, the electrical resistance decreases by 80% of the initial electrical resistance to 2.1 × 10^4^ Ω after heating treatment. Once this value is reached, the time is taken as the alert time for evaluating the sensitivity of the fire alarm.

Besides the above-mentioned fire alarming work, other studies also chose the “trigger electrical resistance” method to calculate the response time, indicating the fire alarm’s sensitivity [60]. It is worth noting that the “trigger electrical resistance” in various systems is not identical, which is closely related to the impedance of the matrix material and the voltage value in an electrical circuit.

### Response Time Based on “Light-on State”

The common heating methods often employed to heat fire-warning samples are a hot plate, furnace, oven, or thermocouple. In addition, alcohol lamps or igniters are also selected to heat fire-warning samples for vivid simulation of early fire-warning processes, which can provide guidance for real warning scenarios. The warning occurrence can be indicated in the simulated fire-warning scene by visually seeing the light-on state. In reference to this phenomenon, the time from light-off state to light-on state can be set as the response time for EFWSs. This methodology for response time corresponds to the “light-on state.”

**Fig. 3:**
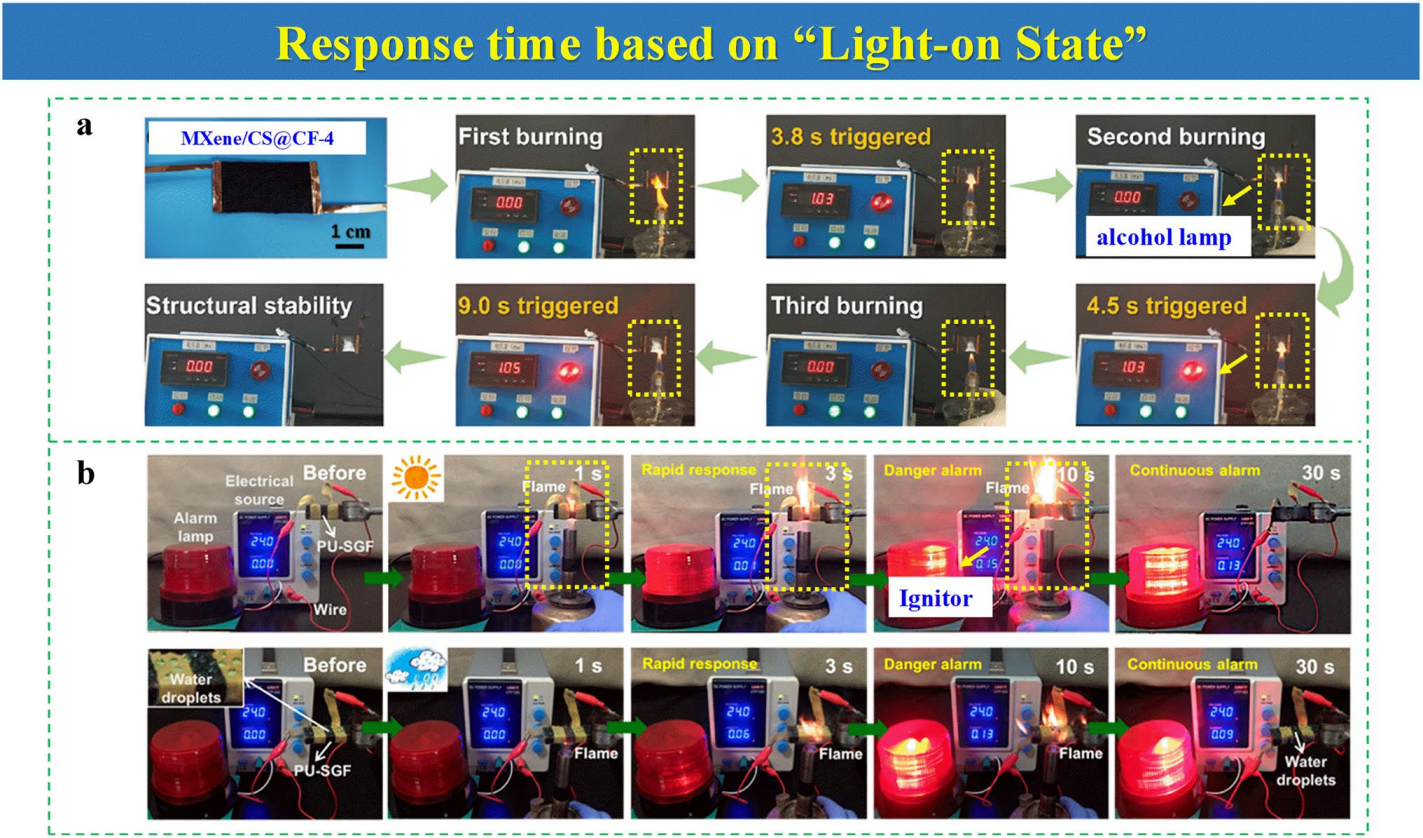
**a** Video snapshots of the fire-warning test process of MXene/CS@CF-4 burned by an alcohol lamp. Reproduced with permission from Ref. [39]. Copyright 2021 American Chemistry Society. **b** Picture from the whole flame detection processes for multilayered PU (PU-SGF) warning sample burned by an ignitor under different environmental conditions. Reproduced with permission from Ref. [64]. Copyright 2018 American Chemistry Society

Response time based on “light-on state” is simple to count the warning time. A smart warning film is a multi-functional composite film consisting of black phosphorene, molybdenum disulfide, and GO. By utilizing an ignitor to burn a warning sample, the electrical resistance decreases dramatically within 1 s, which can induce an alert [61]. As observed in Fig. 3a, the warning can be issued within 3.8 s after a flame attack from an alcohol lamp in a cotton fabric (CF) system by layer-by-layer self-assembly of MXene and chitosan (CS), showing better outstanding flame retardancy and firefighting [39]. Another system based on fire-warning nano-coating that includes GO and functional cellulose coated on combustible materials is fabricated through a simple self-assembly way. After treatment of alcohol lamp, the electrical resistance decreases to implement trigger warning in less than 3 s [62]. It demonstrates that an alcohol lamp can provide enough energy to achieve conductivity conversion. Moreover, one water-based hybrid network including GO nanoribbon, MMT, and polyethylene glycol (PEG) is wrapped in PU foam for a fire alarm, showing a response time of 2 s after burning by ignitor [63]. Besides the alcohol lamp, the ignitor is also applied as an energy source to change electrical resistance. In one silicone-modified PU-based fire-warning system, an ignitor is selected to attack the warning sample (Fig. 3b). When a warning sample encounters a flame attack, the danger alarm can be triggered in a short time of 2–3 s, showing its sensitivity to fire alarm [64]. Besides the mentioned works, there are also other EFWSs in which the response time is calculated in the way of “light-on state” [65]. The “light-on state” method for response times might be influenced by warning lamp power and the surrounding environment.

### Response Time Based on “Light Intensity Change”

Usually, a traditional lamp is used as a warning signal in most EFWSs. After being triggered, the light intensity would change. There is an instrument that can detect the change in light intensity, especially capturing extreme weak light intensity that could not be caught by the human eye. As the above calculation way, this period until the light intensity change captured by the machine can be used as a warning signal, named “light intensity change.”

**Fig. 4:**
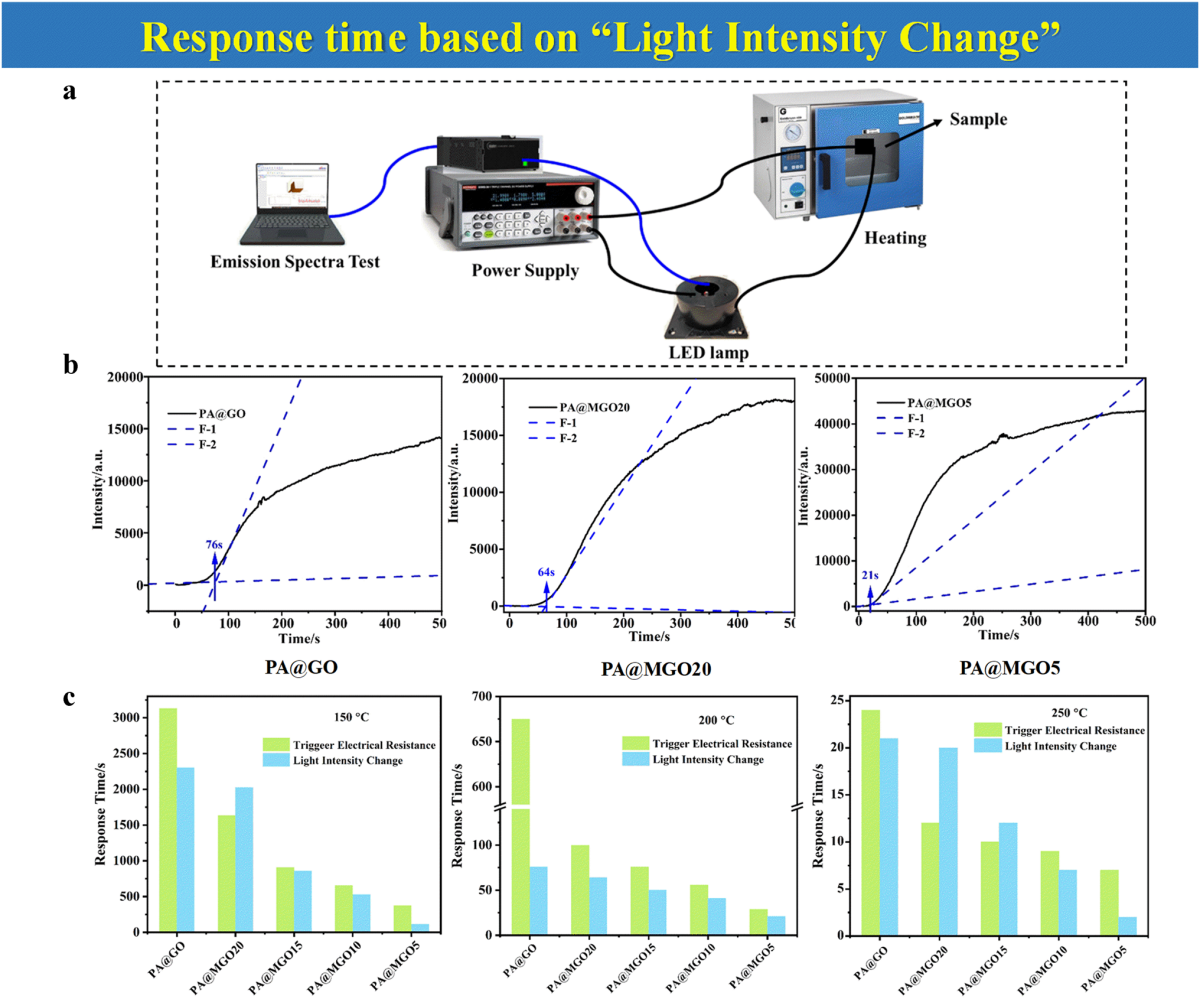
**a** Schematic test process of light intensity change. **b** Doing tangent at lamp intensity change curves of fire-warning samples at 200 °C for response time. **c** Comparison of response time based on calculation methodology between the “trigger electrical resistance” and the “light intensity change” at different test temperatures. Reproduced with permission from Ref. [66]. Copyright 2022 Elsevier

In the work of cellulose paper-based fire alarm study, which is modified by phytic acid (PA), GO, and MXene solution in variable concentrations to prepare fire-warning samples of PA@GO, PA@MGO20, and PA@MGO5, response time based on the “light intensity change” is proposed [66]. The schematic test processing is shown in Fig. 4a. After the materials are exposed to high temperatures, the LED lamp is switched on within a short time, and the corresponding light intensity is recorded. When fire-warning samples are used in an electrical circuit, the real-time light intensity change curves are obtained and shown in Fig. 4b. By making the tangent of the curves, the critical point where the light intensity change appears is used to calculate the response time for an EFWS. This method for response time via quantified light intensity curve has its uniqueness, presenting its advantage of avoiding the visual differences in weak light signals caused by the traditional lamp warning signal.

The comparison between response time based on “trigger electrical resistance” and response time according to “light intensity change” is displayed in Fig. 4c. The response time obtained by “light intensity change” is generally lower than that obtained by “trigger electrical resistance.” This could contribute to the sensitivity of quantified light signals. Even faint light changes can be detected, while they cannot be observed by human visual limitations. Moreover, the selected trigger electrical resistance values in different EFWSs are inconsistent. The trigger electrical resistance set in each system is the one than can ensure the formation of a circuit pathway in one fire alarm, but it is not the exact minimum trigger electrical resistance value. As a result, this might cause a difference in the corresponding response time.

### Response Time Based on “Voltage Change”

The combination between EFWSs and the thermoelectric effect (TE) becomes gradually popular. The fire alarms based on TE are sensitive to the external temperature change, resulting in the change in voltage that is the crucial point to realize the warning. Therefore, the response time can calculate on the basis of the voltage change, which can be named “voltage change.”

**Fig. 5:**
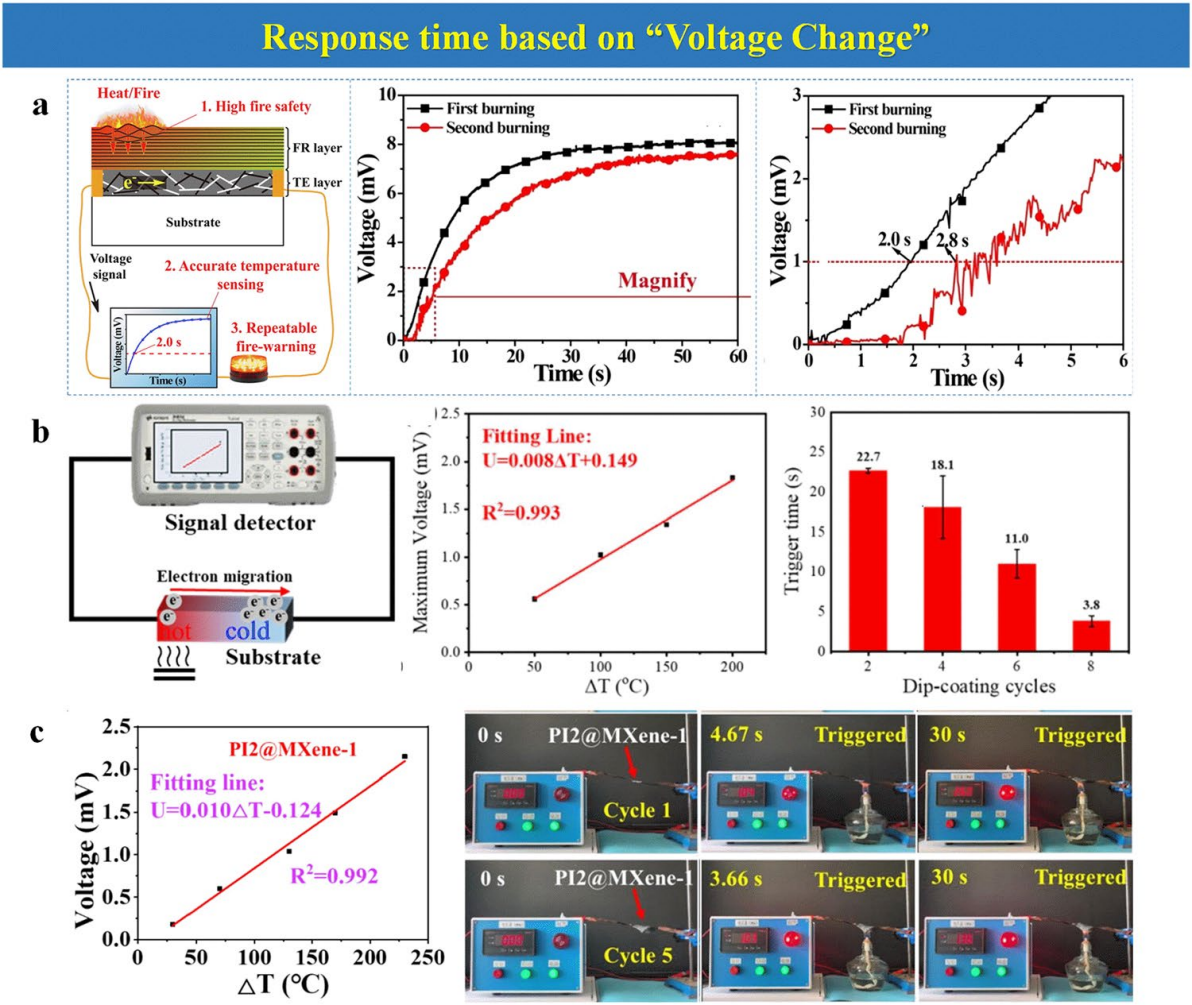
**a** Schema illustration of a fire-warning nano-coating; output voltage curve over heating time in the first and second heating test. Reproduced with permission from Ref. [55]. Copyright 2021 Elsevier B.V. **b** Scheme illustration of thermoelectric effect. The relationship between voltage and temperature difference. Reproduced with permission from Ref. [67]. Copyright 2022 Elsevier B.V. **c** The trigger time of matrix with different dip-coating cycles. Reproduced with permission from Ref. [35]. Copyright 2022 Elsevier B.V

A fire-warning nano-coating that comprises a flame-retardant (FR) layer and thermoelectric layer coated on the matrix is prepared [55]. Because of the TE ability of the sample, the voltage of the fire-warning system gradually increases over heating time. When the voltage reaches 1 v, it is considered that the warning has been triggered. According to “voltage change,” the calculated response time for this system is about 2 s (Fig. 5a). A substrate coated with another fire-warning nano-coating by the layer-by-layer assembly is also a fire alarm [67]. The voltage change can be recorded after heating or burning treatment on one side of the sample. The temperature difference between the two sides of the sample is the key to forming the voltage. In this respect, the relationship between the maximum voltage and temperature changes is established, shown in Fig. 5b. The corresponding voltage value can be obtained through this relationship when the sample is heated to a specific temperature. Furthermore, one fire-warning aerogel prepared by polyimide (PI) and MXene also utilizes the thermoelectric effect to realize fire warning [35]. The relationship between voltage and temperature in this system shows a slight difference from the above one (Fig. 5c). This might be related to the difference in the TE ability of different materials.

In EFWSs without an external power supply and working on TE, calculating response time on “voltage change” is the preferred way. Theoretically, this response time calculation is highly associated with the TE capability for materials and the detection capability of the instrument.

## Thermosensitive EFWSs

Currently, most EFWSs are to detect the temperature change in a pre-combustion stage. Therefore, this section shortly provides a review of EFWSs with different working mechanisms that can implement warnings in smart devices.

### GO-Assisted Fire Alarms

GO has been primarily viewed as a preferred candidate for early fire alarms because of its thermally unstable chemical activity. It can produce conductive reduced graphene oxide (rGO) after experiencing reduced treatment and exothermic disproportionation reaction [47]. In addition, the unique amphiphilic of GO can achieve functionalized hybrid carbon materials with various performances [47].

**Fig. 6:**
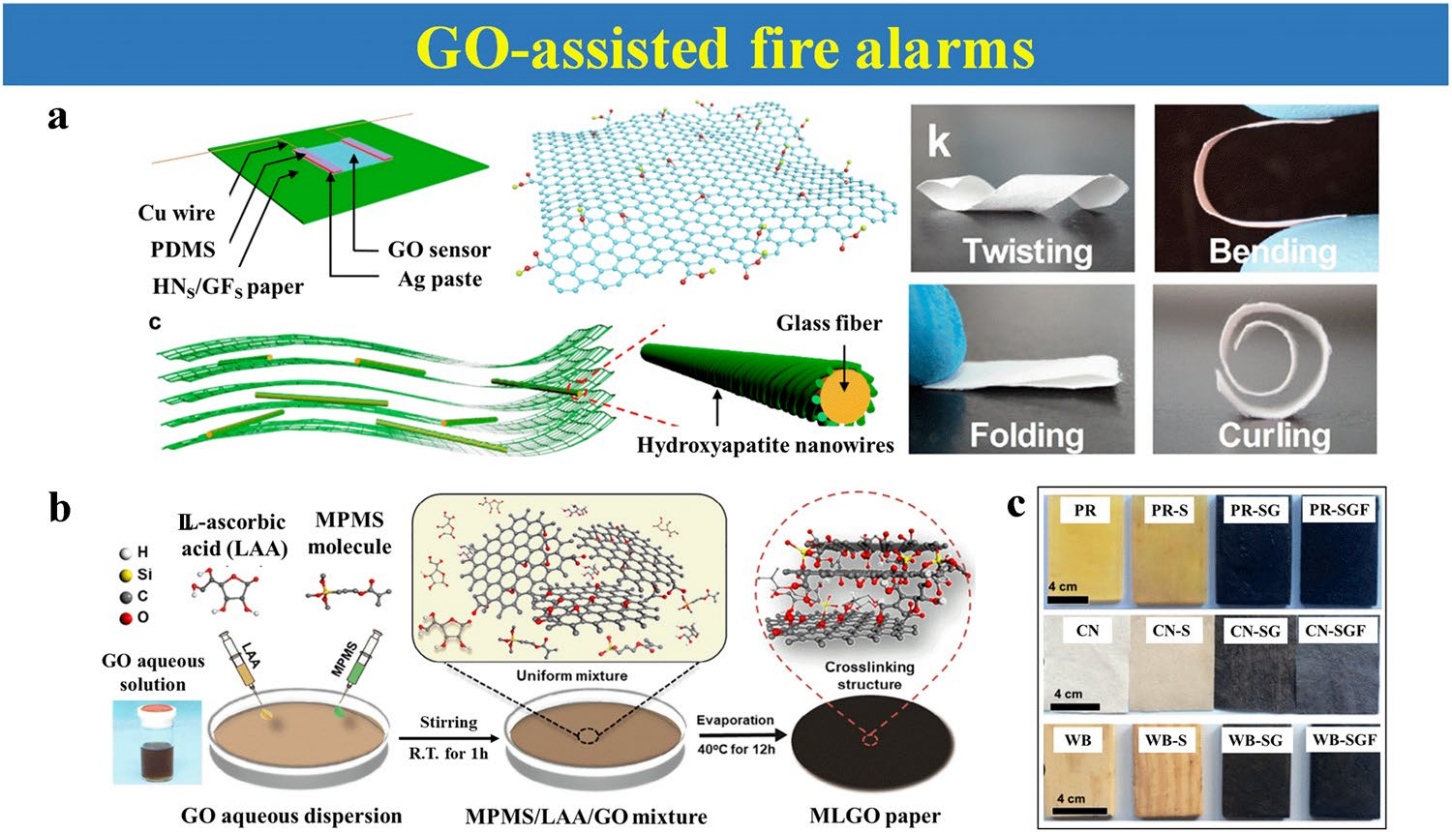
**a** Schematic fabricating process and micro-structure of fire alarm wallpaper, GO structure with rich oxygen-containing groups, and glass fiber paper with multilayered structure. Reproduced with permission from Ref. [80]. Copyright 2018 American Chemistry Society. **b** Schematic illustration of MPMS/LAA co-modified GO (MLGO) paper, MLGO paper. Reproduced with permission from Ref. [83]. Copyright 2020 Elsevier. **c** Photographs of the fire-warning coatings on the surface for different flammable materials: polystyrene resin (PR), cotton (CN), and wood block (WB). Reproduced with permission from Ref. [64]. Copyright 2018 Elsevier

On the basis of the superiority of GO, varied early fire sensors based on different substrates are reported [60, 68–79]. Eco-friendly paper is one of the substrates. As shown in Fig. 6a, a smart fire-warning wallpaper produced by modification of hydroxyapatite nanowires (HNs) and polydopamine-modified GO (PGO) utilizes the conversion of electrical conductivity of GO for warning. With temperature increasing, GO becomes conductive to trigger the warning lamp and warning buzzer at 126.9 °C to warn [80]. Moreover, a kind of wood pulp paper (WPP) coated with phenoxycyclophosphazene-functionalized graphene oxide (FGO) and chitosan-functionalized carbon nanotubes (CNTs) also uses GO to implement early warning in several seconds, exhibiting its sensitivity to temperatures [81].

Besides the environmental paper, a variety of GO-based film materials are fabricated as fire alarms. A GO-based composite film with synergetic benefit from 3-methacryloxypropyltrimethoxysilane (MPMS) and L-ascorbic acid (LAA) is caused by self-assembly phenomena at interfaces of mixture solution. The formation of the film is attributed to the amphiphilicity and surface chemistry of GO [47]. The MLGO paper can show an ultrafast early warning response within 7 s at 300 °C (Fig. 6b). Another hydrophobic sisal cellulose microcrystal film is also described as a fire sensor. This composite film is co-modified by graphene nanosheets (GN), soy protein isolate (SPI), sisal cellulose microcrystals (MSF-g-COOH), and citric acid (CA) with improved flame retardancy, exhibiting sensitive response capability after being burned [82].

Benefiting from the self-assembly ability of GO, a multi-functional mixture solution can be pre-designed to be wrapped into different substrates by typical dip-coating or evaporation methods. This simple, low-cost sample preparation method displays universality to different substrate materials, such as wood black, PU foam, cotton fabric and polystyrene resin (PR). Wu et al. reported hierarchical fire-warning coatings created by GO and silicone structures that can be covered onto PR, wood block, cotton, and PU foam [64]. As depicted in Fig. 6c, the surfaces of flammable materials with a warning layer become black. This resulting coating can not only improve flame resistance of flammable substrates, but also provide a warning signal in 2–3 s.

**Fig. 7:**
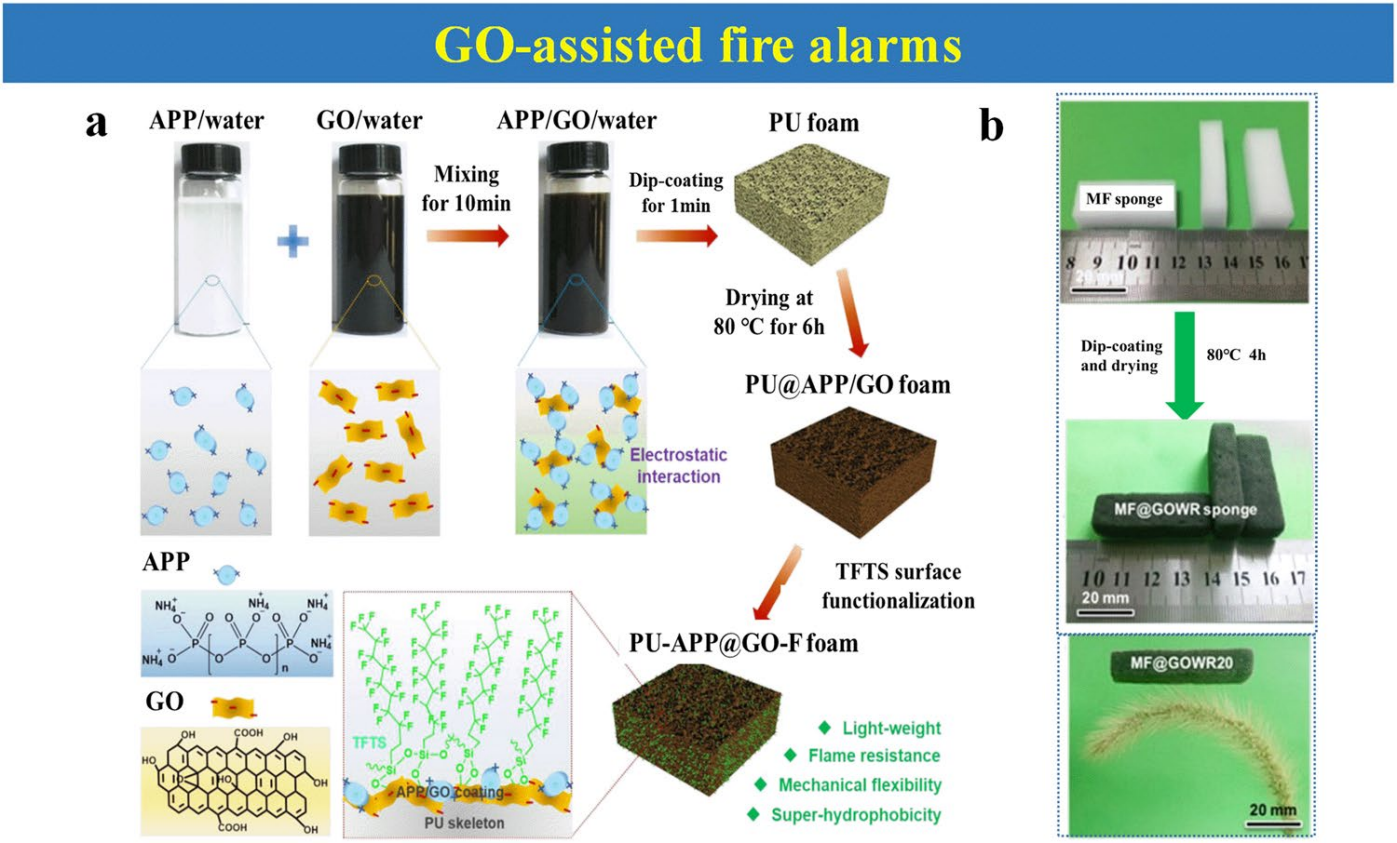
**a** Detailed preparation process of water-based hybrid ammonium polyphosphate (APP) and GO mixture solution coated on the surface of PU foam. Reproduced with permission from Ref. [84]. Copyright 2020 Elsevier. **b** Pictures of pure MF and MF@GOWR sponges via dip-coating way; image of MF@GOWR20 to show lightweight feature. Reproduced with permission from Ref. [18]. Copyright 2019 Elsevier

Apart from the above fire-warning layer, there is another kind of warning layer decorated on the surface of PU foam. The pre-designed water-soluble solution containing hybrid ammonium polyphosphate (APP), silane, and GO can be wrapped on the PU surface for early fire sensor (Fig. 7a). By exploiting GO’s electrical conductivity, this special hybrid coatings can respond to flame within only 2 s and a high temperature of 300 °C at 11.2 s [84]. Other melamine–formaldehyde (MF) sponges can also be used as an early warning substrate (Fig. 7b). In one multi-functional GO wide-ribbon (GOWR)-wrapped MF sponges, both hydrophobic, reversible compressibility, and flame-retardant properties have been improved. Under the fire attack, this alarm is triggered with an ultrafast alarm response of 2 s [18].

As above-mentioned fire alarms in the previous section, a variety of GO-assisted early fire alarms on different flammable materials are proposed [54]. Nonetheless, GO-based early fire sensors are not exempt from limitations in application. They still have some challenging parts that need to be advanced. Firstly, they cannot be used repeatedly and cannot detect fire revival due to the irreversibility nature of the GO reduction. Secondly, GO-based fire sensors require an external power source to generate an electrical signal [35, 39, 55], which will cause inconvenience in operation, ultimately limiting the large-scale application [43].

### Semiconductor-Based Fire Alarms

Except GO, other materials have been proposed for fire detection and thermal sensing, containing semiconductors, conjugated polymers, carbon-based nanomaterials, sulfides, and nano-hybrids [38, 85]. Traditionally, different semiconductors have been carried out for fire detection by utilizing the characteristic of conductivity changes with temperatures. Several semiconductors, such as metal oxides (MOs) or CNT, are well known for gas sensing [86, 87]. Recently, these semiconductor materials have been considered for thermosensitive early fire sensors [40, 88, 89]. Their working principle is similar to the previously discussed GO-based EFWSs by means of electrical resistance change upon heating or burning exposure.

There is a growing demand for flexible fire alarms that can be either integrated into flexible fabric products or used directly as functional fabric products, such as textile, CF, or polypropylene (PP). In fact, semiconductors arise as a potential candidate for fire alarms with the motivation of their excellent sensor characteristics in terms of sensitivity and response/recovery time observed in gas-detecting sensors [87], temperature, or pH sensors [36], as well as their easy implementation within these substrates. The combination between semiconductors and polymers offers notable advantages since this allows creating the low-cost, large-scale, and multi-functional sensors. Concerning fire sensing, different semiconductors have been considered for enhancing thermosensitive properties to achieve sensitive temperature detection, including different MOs such as Fe_3_O_4_ [38], ZnO [36], and SnO_2_ [37]. A sandwich-like fire alarm fabric (Ag@Fe_3_O_4_-MS) based on 1D-Fe_3_O_4_ nanowire arrays and fish-scale-like Ag sheets, designed by in situ layer-by-layer assemblies on the surface of PP nonwoven fabric, is provided [38]. Ag@Fe_3_O_4_-MS is an electrical insulator under room conditions and turns into a conductor when exposed to flame or to an increase of temperature (even below 100 °C). This structure shows a rapid response time of 2 s and is found to be reliable repeatability with at least 15-min alarm time in the flame [38].

**Fig. 8:**
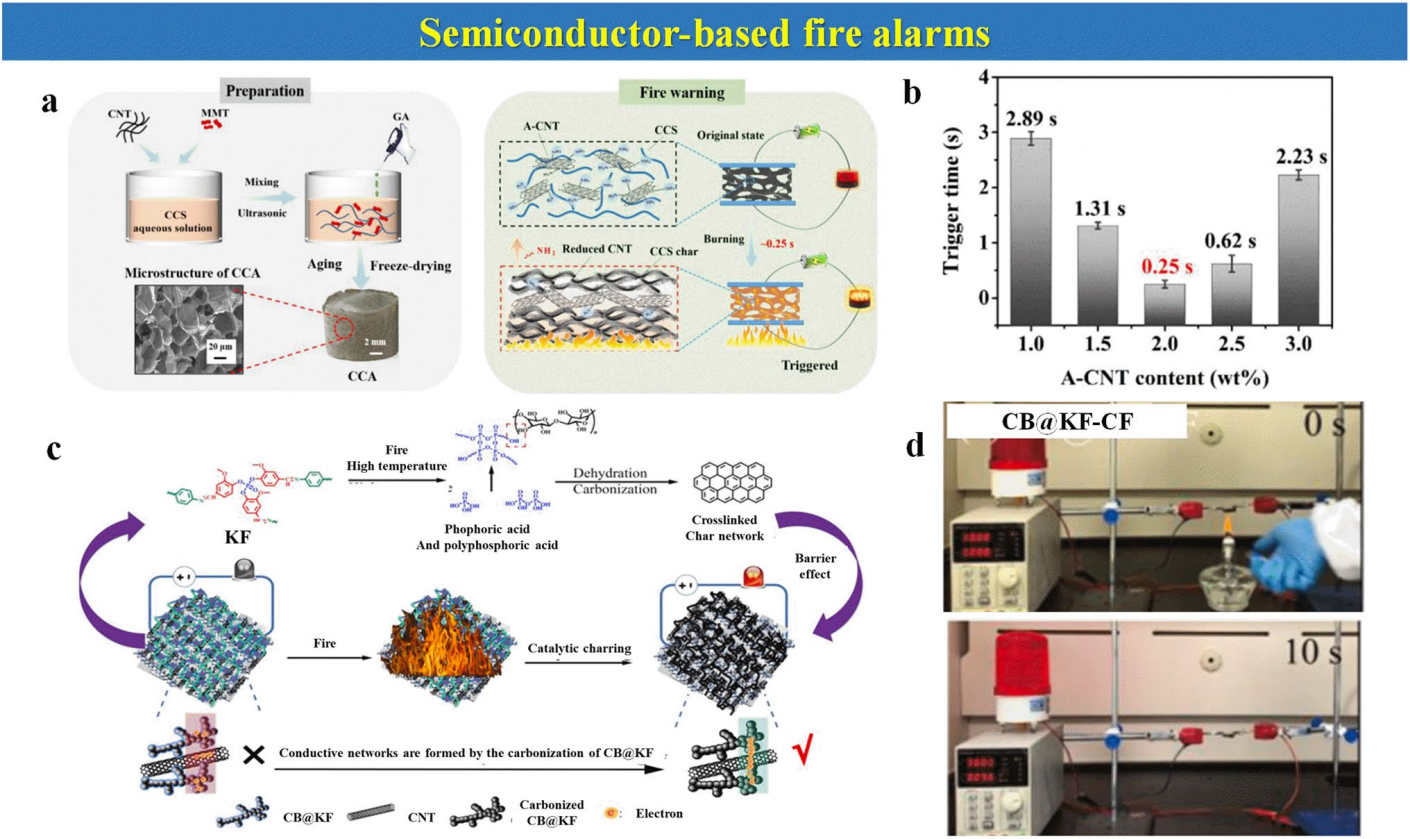
Fire alarms based on semiconductor materials. Design strategy for CCA. **a** Preparation route, supersensitive fire-warning capability, and mechanism of CCA, **b** Fire-warning trigger time of CCA with different A-CNT content. Reproduced with permission from Ref. [42]. Copyright 2020 Elsevier. **c** Schematic flame-warning mechanism of CB@KF-CF. **d** Simulated fire-warning process CB@KF-CF. Reproduced with permission from Ref. [43]. Copyright 2022 Elsevier

Carbon-based allotropies, alongside GO, have also been considered for thermosensitive fire alarms due to several temperature-induced phenomena which can serve as objects to be detected, such as material volume expansion, magnetic susceptibility change, and resistance exchange. As a representative of carbon-based materials, CNT can be employed to prepare an EFWS by monitoring environmental temperature changes [86]. The chitosan/montmorillonite/CNT composite aerogel (CCA) is assembled and shown in Fig. 8a [42]. In particular, the employed amino-functionalized CNT (A-CNT) can provide CCA with high electrical resistance (sharp decrease with increasing temperature), high mechanical strength (to create resistant aerogel), and tubular structure (which acts as an exposed pathway to fire). The combination with carboxymethyl chitosan (CCS) can be a promising substitute for traditional friendly flame retardants due to the abundance of hydroxyl groups and amino groups, endows CCS aerogel with excellent charring capability and thermal insulation. This EFWS can realize a short response time (~ 0.25 s) and could resist a high-temperature flame up to 1200 °C as observed in Fig. 8b.

Another carbon-based carbon black (CB) material for fire sensors has been proposed. It is a novel flame-retardant coating including CB nanoparticles (denoted as CB@KF) blended with polyvinyl alcohol (PVA) and CNT over CF to assemble a fire-warning sensor (CB@KF-CF) [43]. In Fig. 8c, the synthesis and behavior of a fire alarm under fire are summarized. This coating provides excellent flame retardancy for fire sensors because of its carbonization properties. CB@KF-CF exhibits an alarm time under flame in 4 s and at a temperature of 350 °C in 8 s, as observed from the screenshots in Fig. 8d. The shell of CB@KF at a high temperature begins to degrade, which generates some phosphoric acid and polyphosphoric acid to promote the carbonization of CB@KF and dehydration of the substrate. The formation of a highly connected char layer, with a high degree of graphitization, can connect the CNT and carbonized CB@KF to form a continuous and conductive network to trigger the fire-warning system.

Transition metal carbides (Ti_3_C_2_T_x_, MXene) have attracted increasing interest in many applications because of their large specific surface areas, favorable electrochemical properties, and excellent strengths [90]. Recently, some EFWSs about MXene have been proposed, as it possesses fantastic flame-retardant effects accompanied by its high electrical conductivity. Therefore, special attention has to be awarded to this particular subset of materials. An EFWS based on PEG or polyvinyl pyrrolidone (PVP) decorated with MXene has been reported [91]. Under flame, the electron excitation of the titanium network can trigger a resistance transition from an insulating state to a conductive state, showing an ultrafast fire response. Due to silane functionalization, the coatings are reusable and weather resistant, which is fundamental for outdoor applications of sensors. Although MXene-based EFWSs have been proposed based on an electrical resistance mechanism, their combination with other materials has also opened the door for other triggering mechanisms based on the generation of voltage due to the thermoelectric effect, which will be described later.

### Thermochromic-Based Fire Alarms

**Fig. 9:**
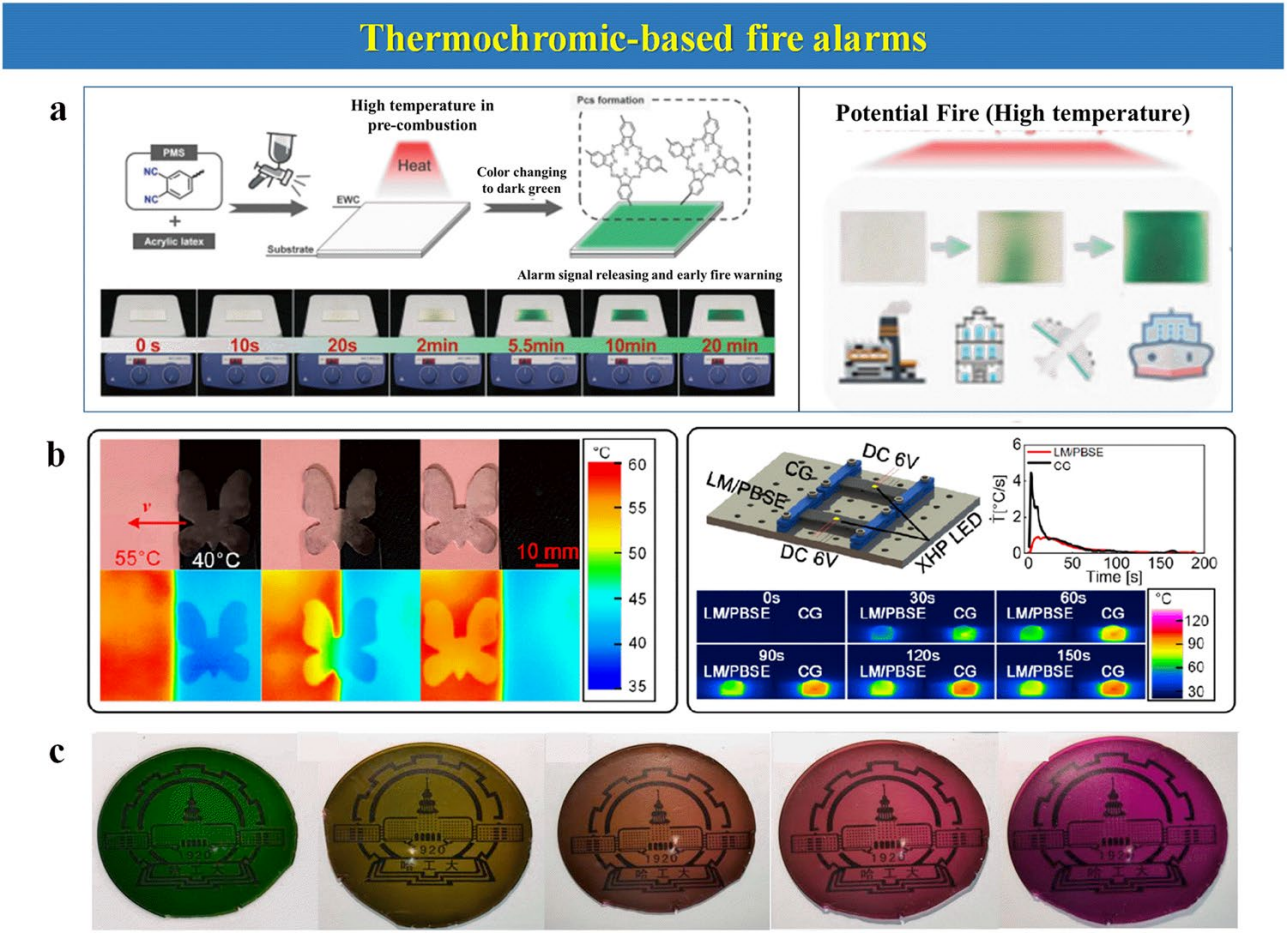
**a** The fabrication of EWC, its color change process, and the potential fire simulation experiments at 275 °C and (fire alarm remote monitoring). Reproduced with permission from Ref. [57]. Copyright 2019 Wiley. **b** The thermal camouflage and thermal dissipation behavior of the LM/PBSE. Reproduced with permission from Ref. [92]. Copyright 2022 American Chemistry Society. **c** The color changes of the PMF material at different temperatures. Reproduced with permission from Ref. [93]. Copyright 2022 Elsevier

Not only the resistance and the generation of a voltage, but also color changes can be applied as possible outputs under the presence of temperature changes. Actually, some materials that display a shift in color under different temperatures or heat accumulation are referred to as thermochromic materials. For instance, the color change can be a response to temperature changes owing to the special changes in the molecular structure. An example is presented by Zhao et al. [92]. After heating liquid metal microdroplets (LM)/polyborosiloxane elastomer (PBSE), benefiting from the tremendous thermal diffusion, the color quickly changed from black to pink at 55 °C (Fig. 9b). This color change caused by the structure demonstrates a significant advantage, especially as a low-temperature warning signal for an EFWS. The phthalonitriles could be transformed to phthalocyanine (Pcs) at about 180 °C, reflected in the color change at a temperature below the combustion temperatures [57]. The earlier fire-warning component (EWC) based on spray-coated phthalonitriles that can transform to Pcs shows a precise color conversion from white to green with increasing temperature, as observed in Fig. 9a. To detect the color change, an intelligent image recognition algorithm is employed, which can display a warning signal within 20 s at 275 °C and 3 s under a real fire. This setup is based on the apparent color change to green with temperature, which is observed clearly by the changes in the FTIR spectra. With the increase in these characteristic modes, the algorithm can change from a 0 to 1 status to display a warning signal. A kind of reversible thermochromic POSS (polyhedral oligomeric silsesquioxane)-metal films (PMFs) with variable ratio of POSS and metal salt Cr(NO_3_)_3_·9H_2_O has been developed recently [93] (Fig. 9c). Due to the temperature increase, ligands such as H_2_O and NO_3_^−^ are decomposed and released which can cause the color change, which is recovered afterward by capturing water molecules from the air and the subsequent re-coordination with PMFs. Thermochromic-based EFWSs are in an early stage of development, although they offer an alternative method to detect a fire at an early stage.

### Shape Change-Based Fire Alarms

**Fig. 10:**
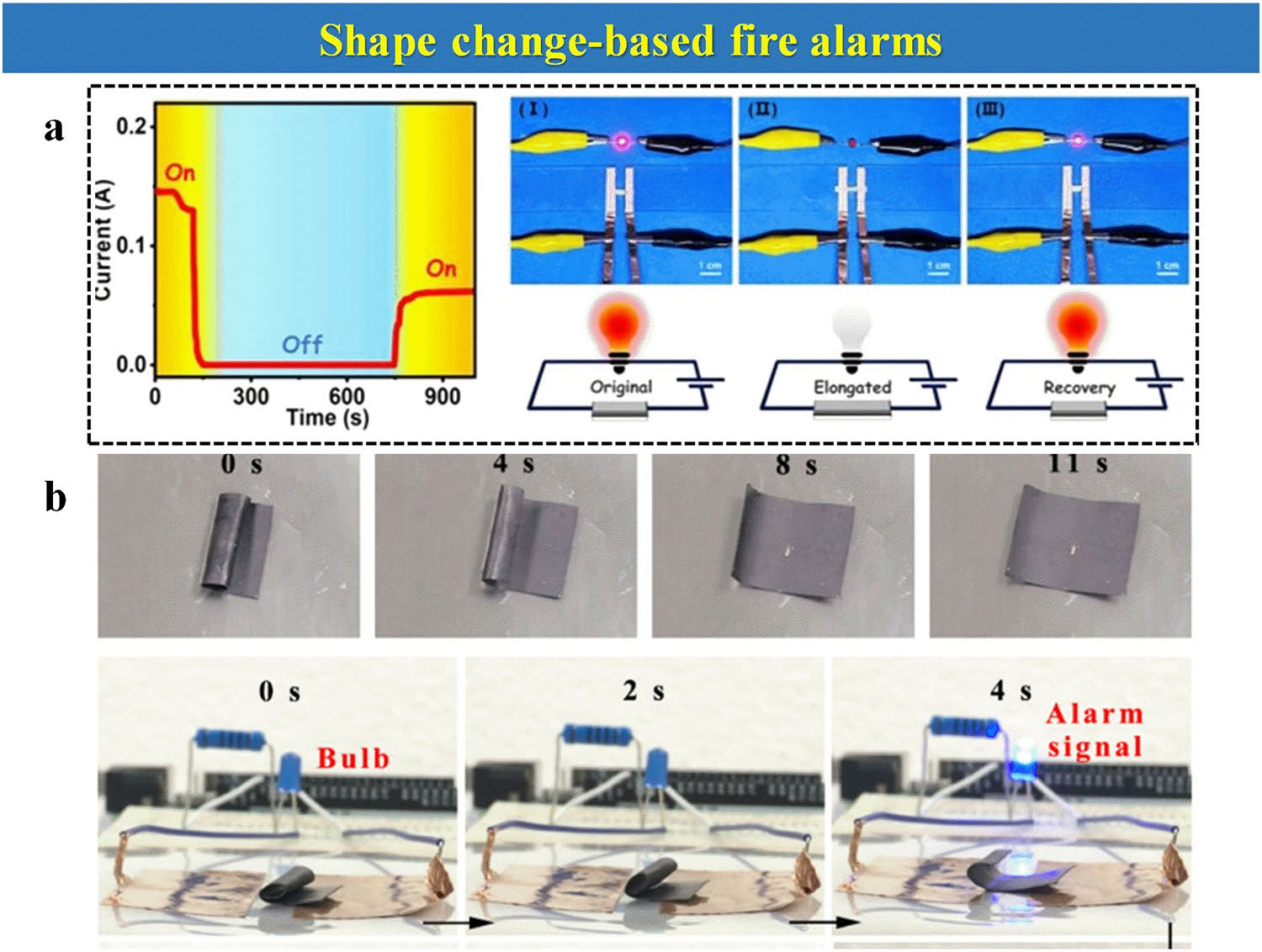
**a** Real-time monitoring of current changes during stretching and shape recovery process via float device and electrochemical workstation at a given voltage of 0.1 V. and Using LED indicators to evaluate the conductivity of sample in its initial state, stretched state, and shape-restored state. Reproduced with permission from Ref. [95]. Copyright 2022 Elsevier. **b** The shape recovery process of rolled-up SMPU/MXene paper and the application as an EFWS. Reproduced with permission from Ref. [58]. Copyright 2021 Elsevier

Shape memory polymers are polymers whose shape can be either permanent or temporary, which can be switched under different circumstances, such as polymer phase transition or changed temperature. Jia et al. [94] fabricated an off-to-on shape memory polymer that can respond to a fire due to the cross-linked polycaprolactone network and its shape memory. This shows from non-conductive at low temperatures to conductive at high temperatures, respectively. The device’s electric current changes during these shape transformations and can be monitored, as observed in Fig. 10a. The sample can be in either the initial state, stretched state, or shape-restored state. Only in the stretched state, the LED light can be turned on to indicate warning danger. In addition, one MXene-based thermoplastic polyurethane (SMPU) fire alarm system is fabricated, which can reveal superior fire retardancy and rapid self-extinguishing performance [58]. This SMPU/MXene paper is rolled up initially. However, the sample becomes flat after heating to form a conductive pathway within 10 s, leading to a trigger to a warning responsive sensor that is observed in Fig. 10b.

### Thermoelectric-Aid Fire Alarms

Thermoelectric (TE) materials have the ability to transform thermal energy into electricity via the Seebeck effect [96]. This distinctive property of TE material provides an alternative mechanism for fabricating EFWSs, by converting thermal energy into a voltage to activate a fire alarm during the pre-combustion stage. Traditional TE materials account for solid inorganic semiconductors (Bi, Te, Se, and their based alloys [97, 98]), which benefit from excellent TE performance. However, these materials are typically expensive, scarce, toxic, and lack flexibility. Recently, due to their high thermopower, ionic conductors, such as liquid ionic conductors and gel ionic conductors, have emerged as an alternative TE material [99]. Furthermore, conductive polymers (CPs) are also promising TE materials. In particular, PEDOT:PSS (poly(3,4-ethylenedioxythiophene):polystyrene sulfonate)-based composites have demonstrated their high potential as TE material [97, 100–102]. To obtain a higher-voltage output, TE materials are often combined in arrays containing p- and n-type TE materials to create thermoelectric generators (TEG), and some systems even work without external power supply [44, 103]. It is worth mentioning that one of the main advantages is the good reversibility of TE-based EFWSs, resulting to the repeated usage for assembled EFWSs, opposed to the irreversible reduction of GO-based systems.

**Fig. 11:**
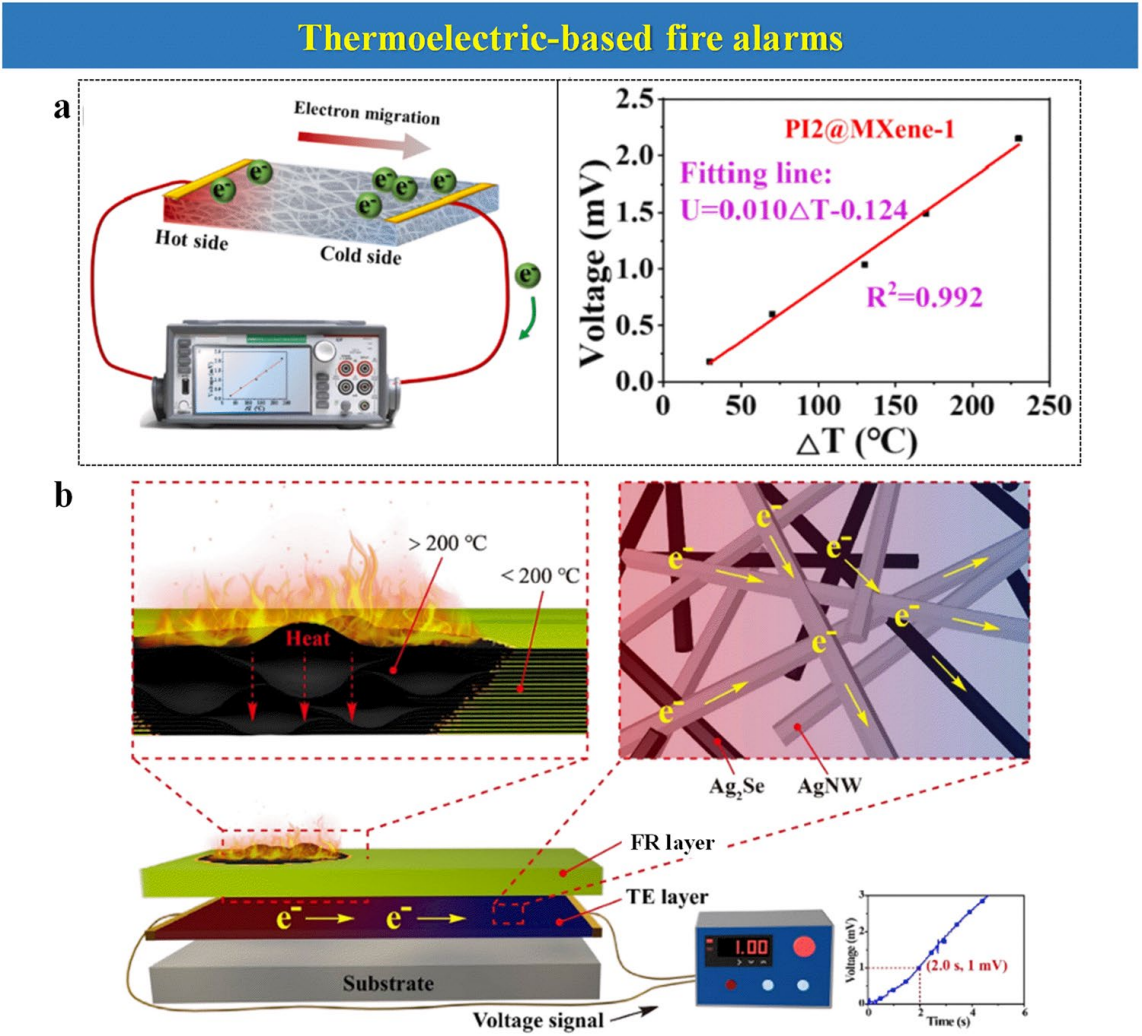
Typical relevant TE-based EFWSs: **a** The schematic diagram of a temperature sensing for PI@MXene and the liner fitting curve of corresponding voltage vs temperature difference. Reproduced with permission from Ref. [35]. Copyright 2022 Elsevier. **b** Structural illustration of TE-based EFWS. Reproduced with permission from Ref. [55]. Copyright 2021 Elsevier

To understand the TE-based EFWSs in practice, an instance is discussed. As shown in Fig. 11a, the left section of PI/MXene (polyimide/MXene) composite is heated (the hot side) as compared with the opposite side, which is often left at room temperature [35]. The difference in temperature triggers a voltage because of the Seebeck effect, whose magnitude depends on the temperature gradient as observed on the graph in Fig. 11a. Therefore, in the practical case of a fire, the high-temperature difference could generate a voltage warning signal which could trigger a fire-warning system [39]. This alarm could be communicated wireless, which has been particularly interesting recently with the IoT development [104]. Fortunately, the applications of TE-based EFWSs have been proposed as wearable sensors to protect humans [44], detect forest fire [96], or as part of personal protective equipment for firefighters’ clothing [39].

The implementation of TE-based fire alarms has been deployed over the last two years. Wu and co-workers [96] have used ionic liquids 1-ethyl-3-methyl-imidazolium acetate ([EMIm][Ac]), 1-ethyl-3-methylimidazolium bis(trifluoromethylsulfony)imide ([EMIm][TFSI]) to assemble a paper-chip fire sensor. It could trigger an EFWS without external power after encountering a flame. However, the leakage of ionic liquids limits its further application. Additionally, Jiang et al. [35] (Fig. 11a) have proposed the PI@MXene aerogel, driven by the excellent thermoelectric, electrical conductivity, and flame retardancy of MXene. When being burned, the fire-warning response is triggered within 5 s. Owing to the lamellar structure of MXene nanosheets, PI@MXene exhibits fast self-extinguishing within 1 s after exposure to a flame, showing excellent flame retardancy.

Both thermosensitive and strain sensors based on poly(3,4-ethylenedioxythiophene) polystyrene sulfonate (PEDOT:PSS)/CNT/waterborne polyurethane (WPU) have been proposed [44]. PEDOT:PSS acts as a conductive binder of CNT when the composite is stretched. Surprisingly, this sensor shows a quick voltage rise response (∼0.7 s) to a slight temperature difference of 5 K. The composite films can maintain stable TE performance after washing 1000 times and withstand repeated bending and stretching.

The current trends in TE materials are in synchrony with the emergence of new technologies, such as the IoT or artificial intelligence (AI), which has led to a growing interest in wearable thermoelectric generators (WTEGs) and self-powered materials (maintenance-free power sources) [44, 101, 105]. Many applications revolve around the idea of using human body heat and strain to monitor physical signals generated by real-time human activities [44, 106], by creating fibers-based devices into woven yarns, and clothing [107], and even for face masks [98].

An interesting TE-based EFWS has created a special nano-coating by combining a dermis-mimicking TE layer and an epidermis-mimicking flame-retardant (FR) layer (Fig. 11b) [55]. This imitates the human skin, in which the dermis can detect temperature changes and send an electrical signal to the brain. At the same time, the dermis layer is also a fire-resistant layer to protect composite. The dermis-mimicking layer comprises the combination of silver selenide (Ag_2_Se) nanorod, silver nanowire (AgNW), and polyvinyl butyral (PVB), which are spray-coated over a substrate. In contrast, the epidermis-mimicking FR layer is constituted by epoxy silane-modified MMT and carboxymethyl chitosan (CCS). This EFWS presents excellent fire-warning ability within 2 s. It can trigger the fire-warning device within 2.8 s when it is the re-burned and accurately measured temperature between 100 and 300 °C, displaying output voltages within the mV range. Zeng et al. [67] also employ a TE layer based on MXene and an FR layer composed of MMT nanosheet and 2-ureido-4[1H]-pyrimidinone-containing cellulose (UPC) via layer-by-layer assembly, which can display great alarming ability. More importantly, the combined action of the hydrogen bond interactions attributed to UPC exhibits self-healing properties that could recover within 24 h of damage, showing the multifunction of this system.

Another advantage of TE-induced voltage besides powering fire alarms is that they can harvest low-quality heat from industry waste, automobile exhaust, and other unexploited heat energies [96]. In fact, in the scenario of a fire, the vast quantity of energy produced could be recycled and utilized to promote fire suppression and pollution reduction [108]. Deng et al. have proposed the waste heat recovery and utilization system, which can achieve an efficiency of the average heat flow utilization of 58% and produce up to 692 W. This idea of fire-energy harvesting can also be used at a home scale, as fire from wood is usually used in rural areas for cooking, and heating can be exploited for micro-scale power generation using thermoelectric generators [109].

Nonetheless, the current TE-based EFWSs are faced with issues that need to be tackled, such as limited preparation methods, complex structural designs, lower operating voltages, strain instability, and limitation of interfacial engineering [44, 105]. Thus, further research needs to be conducted.

### Triboelectric Nanogenerator-Based Fire Alarms

**Fig. 12:**
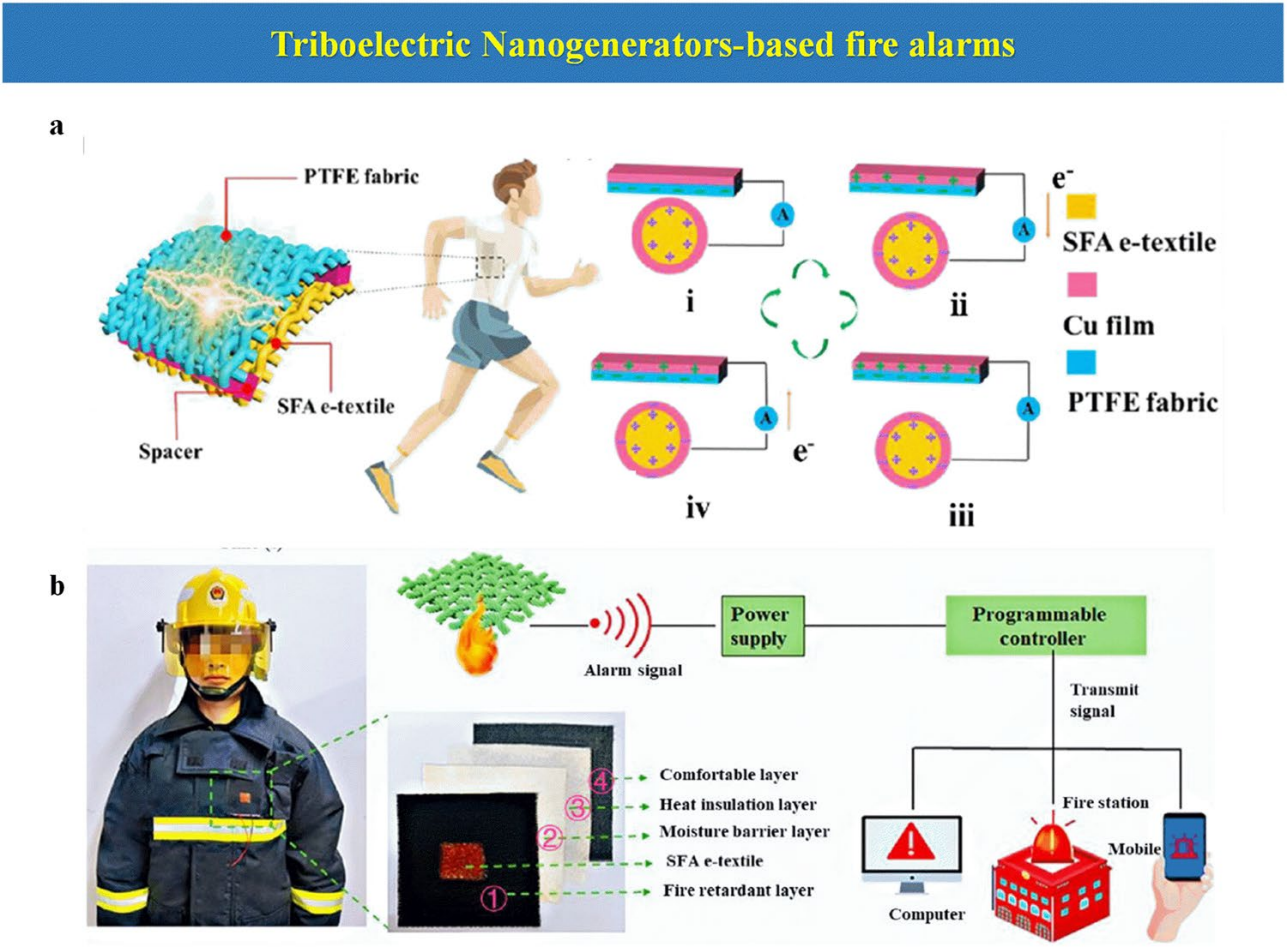
**a** Basic electricity generation mechanism of the TENG by a vertical contact-separation mode. **b** Temperature-response resistance changes the behavior of the SFA e-textile under different temperatures, and the operating mode of the SFA e-textile as a fire alarm material in firefighting protection. Reproduced with permission from Ref. [54]. Copyright 2022 American Chemistry Society

An arising interest has been growing around TENGs for EFWSs. They have been utilized to harvest various forms of mechanical energy from the environment as a sustainable power supply [110]. The operation mechanism of TENGSs is on the basis of the coupling effect of contact electrification and electrostatic induction [111]. A TENG is usually composed of two tribo-materials, a positive and negative and electric contact, as observed in Fig. 12a. The friction produced by an external force between these two surfaces causes the electrons to be transferred from positive to negative materials, which produces a flow of electrons. This electrical current can be harvested and employed by connecting a TENG with an external circuit. One exciting feature for TENGs as EFWSs is that they could gather other energy sources produced either during a fire (wind and smoke) or human movement. Ideally, one main goal for TENGs is that they can be self-powered, i.e., capable of generating their electricity signal as a stimulus response.

One TENG-based EFWS is developed by combining TENGs and a thermosensitive sensor based on a polydopamine-modified GO-based sensor over hydroxyapatite nanowire paper [54]. The force applied by wind waves causes the contact between the copper electrode and a dielectric silicone sphere to generate a voltage signal. Next, the self-generating can switch LEDs on to achieve a warning under the exposure of a flame.

Hybrid energy harvesters, based on TENGs, provide a promising method for energy harvesting as well as fire sensing. Portable and wearable electronics are an emerging field due to the synergistic effect benefiting from the energy-producing during movement. Textiles can work as a substrate for self-powered wearable electronics, where vital research has been aimed toward wearable triboelectric nano-generators [54]. He et al. have fabricated an ultralight self-powered fire alarm electronic textile (SFA e-textile) based on conductive aerogel fiber that comprises calcium alginate (CA), Fe_3_O_4_ nanoparticles (Fe_3_O_4_ NPs), and lightweight silver nanowires (AgNWs), which can work as the triboelectric positive material. The TENG is assembled with the interaction with a triboelectric-negative polytetrafluoroethylene (PTFE)-coated CF. Interestingly, this e-textile allows a synergistic approach by obtaining accurate temperature monitoring and energy harvesting in firefighting clothing. SFA e-textile is integrated as an extra layer into firefighting protective clothing, and sensing different temperatures between 100 and 400 °C is repeatedly measured. This temperature detection could be transmitted, and an alarm sign could be triggered to protect the integrity of firefighters, as observed in Fig. 12b. In addition, a self-powered fire self-rescue location system is further established based on the SFA e-textile that can help rescuers search and rescue trapped firefighters in fire cases, as they could be identified in real time.

Nonetheless, TENGs have disadvantages mostly related to the low output voltage and long-term abrasion resistance, as the repetitive contacts tend to decrease their mechanical durability. They are associated with applications in EFWSs, especially those comprised of fabrics with very low fire resistance. Currently, research is being conducted regarding fire-retardant TENGs [112]. Cheng et al. have developed a textile-based triboelectric nanogenerator (T-TENG) with improved fire resistance by employing a flame-retardant conductive fiber prepared by a simple and effective LBL self-assembly method with combined flame-retardant synergistic systems of phosphorus-nitrogen (polyethylenimine (PEI) and melamine (MEL) as the cationic polyelectrolyte solution and phytic acid (PA) as the anionic electrolyte solution) [113]. Ionic liquid-based TENGs have also shown low flammability, which could be highly interesting for TENGs EFWSs [114].

**Fig. 13:**
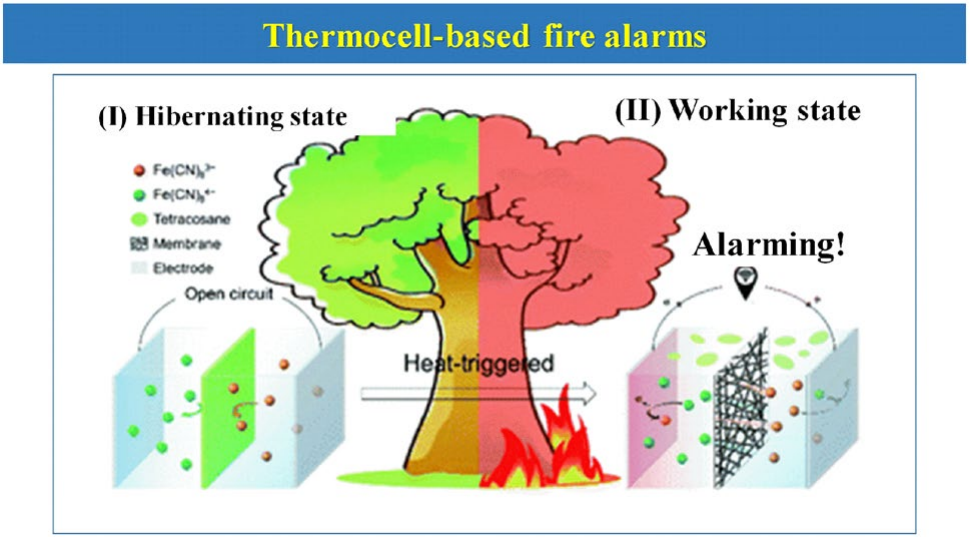
Schematic diagram of the thermocell device in both hibernating and working state. Reproduced with permission from Ref. [115]. Copyright 2021 Royal Society of Chemistry

A special mention should also be given. It is the reported work by the use of thermocells based on the thermogalvanic effect for a low-cost and scalable heat-to-electricity conversion as well as an EFWS system [115]. This effect is mainly based on the temperature dependence within the electrochemical redox potentials. They can produce a high voltage of around 2 V, enough to trigger the alarm system, which could be applied on a forest area (Fig. 13).

Some representative EFWSs in the above categories, in terms of preparation methods, solvent, sensitivity, and multi-functions, are summarized in Table 1. The comparison between them enhances an evaluation of the current achievements and provides potential developing directions.

**Table 1:** A comparative analysis between EFWSs with different working mechanisms

EFWSs	Working mechanisms	Methods	Solvent	Response Temperature/ºC		Response time	Reversibility	Multi-functions	References
				≥ 300	< 300				
MPMS/LAA/GO	Thermal reduction of GO	EISA	Water	√	–	7 s	×	Yes	[83]
FGO/CNT@WPP	Thermal reduction of GO	LBL	Water, acetic acid	√	–	7 s	×	No	[81]
M@GO-PTS10	Thermal reduction of GO	LBL	Water	–	√	1 s	×	No	[116]
CB@KF-PVA-CNT	Semiconductor properties	Dip-coating	Water, dichloromethane	√	–	8 s	×	No	[43]
MMT/chitosan/A-CNT aerogel	Semiconductor properties	Freeze-drying	WATER	–	–	0.25 s	×	Yes	[42]
PMS	Color change	Evaporation	Water	–	√	20 s	×	No	[60]
POSS-Metal films	Color change	Evaporation	Cr (NO_3_)_3_⋅9H2O	–	√	-	√	No	[93]
MXene, SMPU	Shape change	–	Boric acid	-	√	11 s	×	Yes	[92]
PCL, Ag layer	Shape change	–	–	–	√	5 s	×	Yes	[95]
PI@MXene	Thermoelectric effect	FREEZE-drying	Water	–	√	5 s	√	Yes	[35]
MXene, CCS	Thermoelectric effect	Dip-coating	water	–	–	3.8 s	√	Yes	[39]
CA/Fe_3_O_4_ NP_S_ and AgNWs	Thermoelectric/Triboelectric	dip-coating	–	–	–	2 s	√	Yes	[54]

*MPMS* 3-methacryloxypropyltrimethoxysilane; *LAA* L-ascorbic acid; *EISA* evaporation-induced self-assembly; *FGO* functionalized graphene oxide; *CNTs* carbon nanotubes; *WPP* wood pulp paper; *LBL* layer-by-layer deposition; *PTS* phenyltrimethoxysilane; *M@GO-PTS* MXene modified GO-PTS film; *CB* carbon black; *PVA* polyvinyl alcohol; *A-CNT* amino-functionalized carbon nanotube; *PMS* phthalocyanines precursor molecular sensor; *POSS* polyhedral oligomeric silsesquioxane; *SMPU* shape memory polyurethane; *PCL* poly(ε-caprolactone); *PI* polyimide; *CA* calcium alginate; *NPs* nanoparticles. √ means “Yes”; ×  means “No”

## Warning Signals for EFWSs

Warning signals in EFWSs comprise all the different mechanisms of action to show the formation of a fire. Therefore, after the information detection is completed, it needs to be converted into warning signals in one way so that humans can be informed about this issue. Traditionally, these include the ignition of a lamp, a LED light, or any sound produced by an electronic buzzer. In addition, wireless transmission has arisen as a more up-to-date and efficient warning signal way in recent times. In this section, the different warning signals reported for EFWSs will be discussed.

### Traditional Warning Signals

**Fig. 14:**
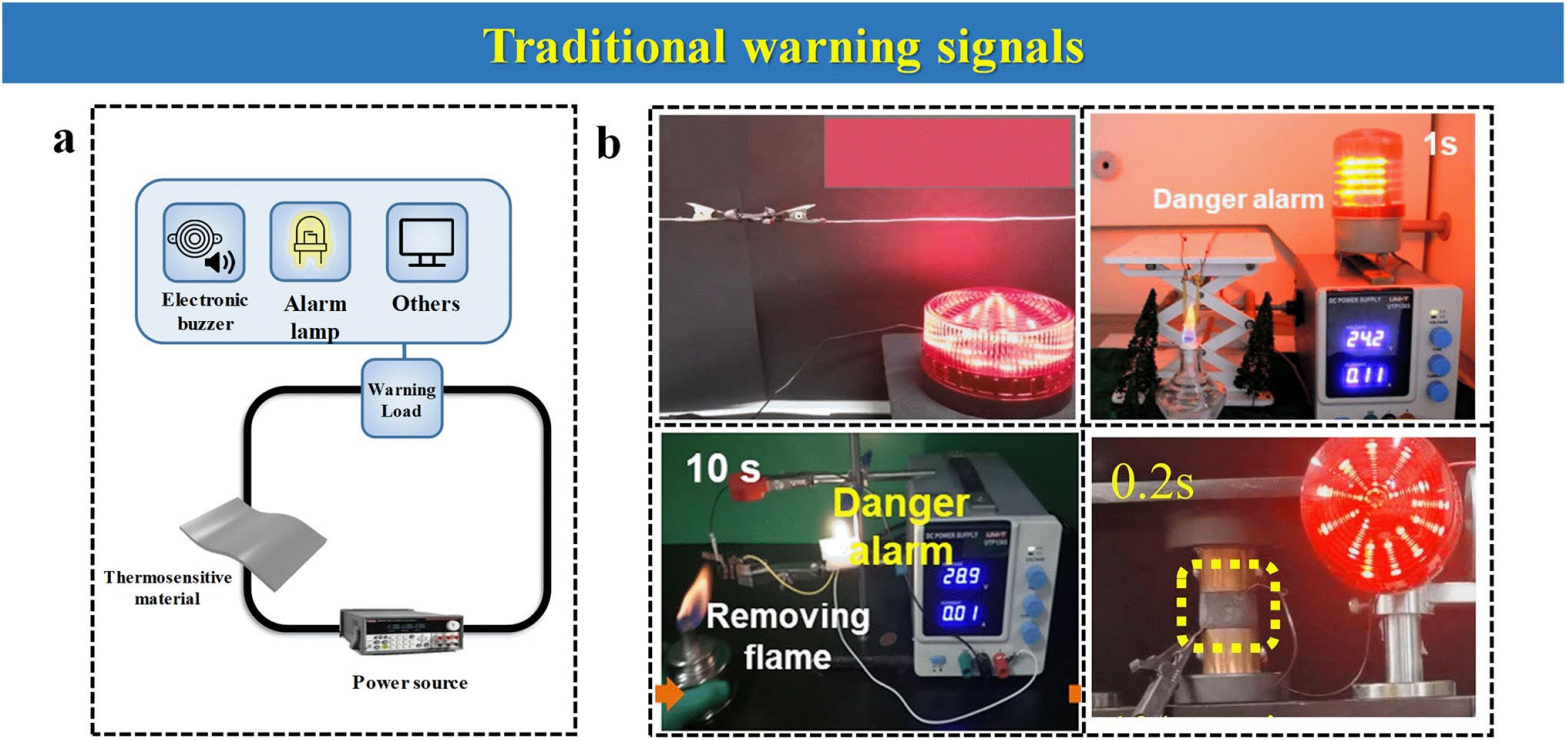
**a** Traditional warning signals comprising alarm lamp or electronic buzzer in fire alarms. **b** Examples of traditional warning alarms. Reproduced with permission from Refs. [40, 60, 69, 88]. Copyright Elsevier

**Fig. 15:**
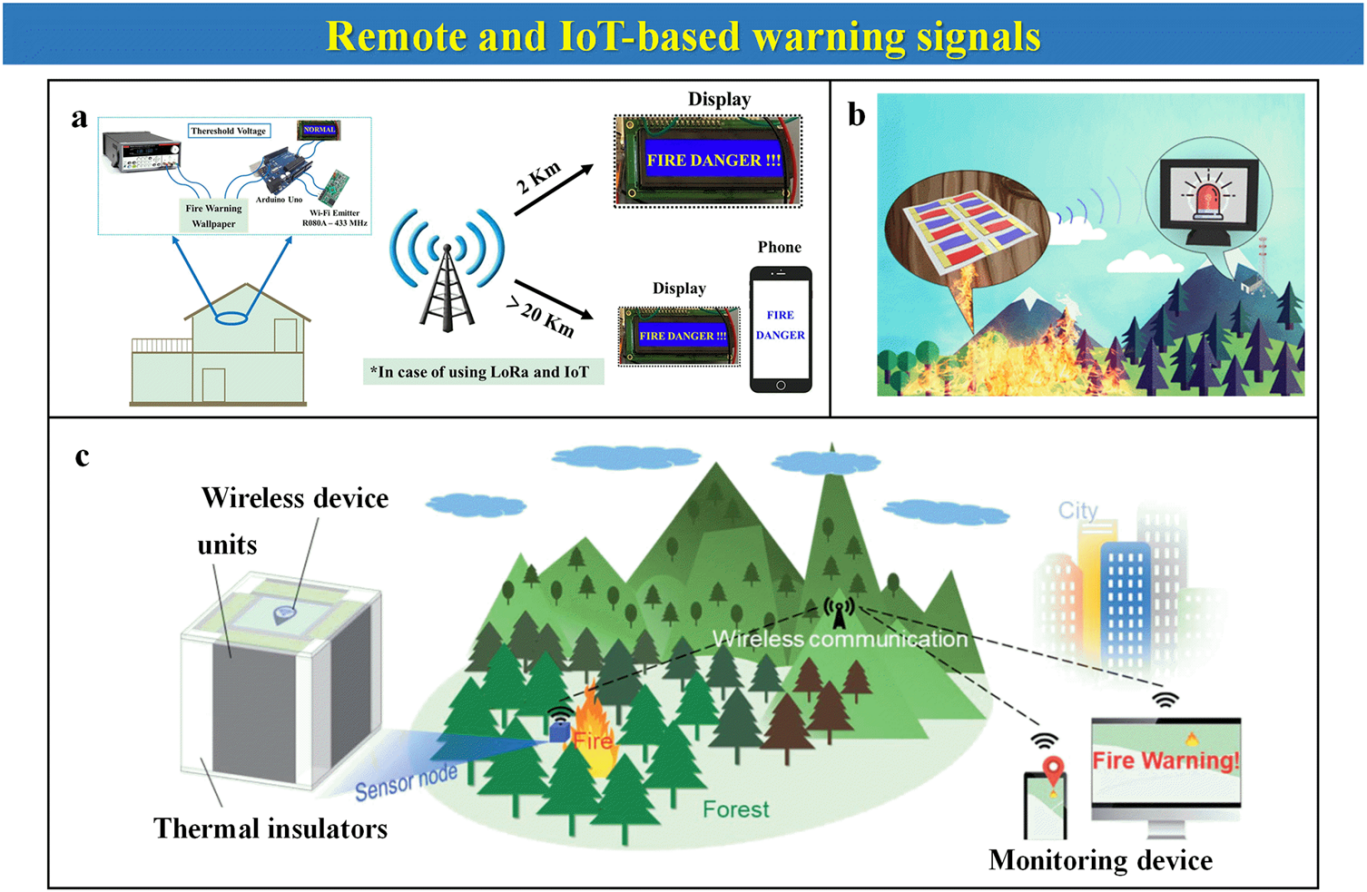
Remote and wireless IoT-based warning systems. **a** Schematic of wireless conversion from fire information to Wi-Fi signal for remote monitoring fire alarm process. Reproduced with permission from Ref. [66]. Copyright 2021 Elsevier. **b** A remote EFWS from a forest. Reproduced with permission from Ref. [96]. Copyright 2020 American Chemical Society. **c** forest fire-warning system based on wireless conversion. Reproduced with permission from Ref. [115]. Copyright 2021 The Royal Society of Chemistry

The common warning signals are based on a simple electrical circuit comprising the sensor material, cables, and power supply, a warning LED, or a lamp (Fig. 14a). When the threshold resistance or the voltage requirements are met, the LED light ignites as the circuit is completed, as observed in Fig. 14b. Other simple electronic devices such as electronic buzzers can be employed for sound alarms. More sophisticated electric devices can be formed outside proof of the generation of this signal. Those involve the combination of an electronic controller. The main drawback of these warning signals, on top of the necessity of a power supply, is that they cannot display off-grid warning signals. This is particularly problematic when the fire hazard occurs at a certain distance (such in the case of a remote forest fire), or when the conditions met on a fire (smoke, dust) can inhibit the visual range of humans and cannot observe the ignition of the LED light. It can hardly provide reliable alarm signal output only due to the traditional warning signal conversion way. For that, the main approach has been the development of wireless and Bluetooth warning signal transmission, as depicted in Fig. 15, which can be remotely sent to any display such as a mobile phone to bring enough time for evacuation.

### Remote and IoT-Based Warning Signals

Several EFWSs either operating locally or/and sending information remotely make use of IoT platforms to monitor relevant physical magnitudes depending on the applications. In the internet era, objects such as temperature, humidity, pressure, and luminosity are often connected with automated systems that can control their values. In addition, physical magnitudes are analyzed in real time or off-line at some distance far from the point of measurement. Connections can be established between people and people, people and things, and things and things [117].

Several IoT-based works can be read in EFWSs [86, 118–130]. Sarwar et al. [118] utilize the change rate of smoke and temperature the same as humidity and gases such as CO to detect the presence of fire. In this respect, DHT22 and MQ-7 gas sensors are used, and as soon as a high probability of fire is detected, a message is sent via GSM to a smartphone. Ramteke et al. have proposed a web-video camera system based on the analysis of flame motion and color present in a fire [119]. Characteristics of the flame can be monitored, and alarm messages would be sent by GSM to a control unit as soon as fire ignition is detected. Merchant and Ahire Gere [120] measure the blade aging of a cutter tool with an LM35 photoresistor, a Hall ACS712 current sensor, and a smoke detector, connecting all of them to a Raspberry Pi, which can analyze the data and send information to a Web site. This work could be used as a fire-warning system too. Because these fire detectors are common ones, all sensing data can be sent to an IoT platform. Dampage et al. have developed a wireless sensor network able to monitor online and simultaneously calculate in each node ratios of relative humidity, light intensity, and carbon monoxide levels in a specific forest area with high efficiency and low energy consumption [122]. DHT22, light-dependent resistor (LDR), and MQ22 sensors can detect these physical magnitudes. Using a machine learning model, if a fire is detected by the array of sensors, a warning message would be sent with the SIM 800 L GSM/GPRS module to operators and responsible authorities to mitigate the possible damage.

Permana et al. have designed a wireless sensor network made of MQ-2 gas, LDR, and LM35 temperature sensors connected to an ESP32 microcontroller that allows monitoring of corresponding physical magnitudes, which could be seen in an Android cellphone [123]. As soon as sensors reach the threshold programmed, a message is sent to cellphone users to advise of the presence of fire. Sapasova et al. [124] fabricated an integrated IoT-based fire alarm system constituted of DHT11 sensors, which allowed measuring temperature and humidity, infrared (IR) flame sensors made of IR photodiodes for detecting the IR radiation coming out of fire flames, smoke sensors to the advice of the presence of fire, gyroscopes to detect ground motions, lights to detect the presence of floods, and loudspeakers to send sound messages of guidance. What is more, RGB LED pixels WS2812B are connected to the gyroscopes for sending alarm light messages in the case of ground motion. This fire-warning system is integrated at different positions of one of the prominent monuments of Varna (Bulgaria) to protect it against fire, earthquakes, and floods by advising the technical staff and general public of its actual status by visualizing these physical magnitudes in the Internet. Xuemei et al. [125] have developed a self-powered intelligent fire detection system (SIFDS) made of a triboelectric nanogenerator (F-TENG) that not only detects the wind direction and strength but supplies energy to the fire detection system. For wind speeds of 4.3 m s^−1^, the electrical power density of 4 W m^−2^ could be achieved by lighting up 100 LEDs and supplying power to a hygrothermograph, the same as sending remotely and sharing through the internet temperature and humidity values by using Bluetooth technology. Based on these monitoring tools, SIFDS is constructed by monitoring the wind direction, the wind speed, temperature, and humidity and predicting the risk of fire according to the fire spread speed and direction. Haryanto et al. have proposed a smart control and alarm fire-warning system to be used in kitchens that allowed monitoring the temperature with the LM35 sensor, smoke with the MQ2 gas sensor, and the presence of humans with microwave sensors by using the ESP8266 Wi-Fi module that allows visualizing online such parameters [126]. In case humans are not present in the kitchen, as determined by the MQ2 microwave sensor, and one of the previous physical magnitudes exceeds a programmed threshold, the LPG lid and the stove might be turned out with an application installed on the user’s smartphone the same as alarm messages of the increase in these parameters. One very similar work is also presented [127]. Here, temperature, humidity, flame, and gas sensors sent values continuously to an Arduino board and activated outputs such as a LED or a buzzer as soon as values higher than programmed thresholds were achieved, indicating a high possibility of fire. In this situation, a water sprinkle is launched by a relay used as a switch to control a motor that pumps water from a tank. An ultrasonic sensor informed about the water level in the tank and the real need of refilling it. All readings from the sensors are displayed on a Web site by using Ubidots and could be displayed continuously on a smart phone.

Detection of CO, flame light, and temperature with CNT is described to fabricate a fire detection sensor [86]. Despite the fact that some improvements should be performed in the CNTs, their use as fire detector sensors connected to IoT will be carried out very soon. Vijóvic et al. present the great potential of Raspberry Pi as a sensor Web node for home automation, more specifically, as an EFWS [128]. Two analog temperature sensors, namely B57045k10 and 10Kntc, are connected to an analog digital converter (ADC) and this one to the raspberry via the I2C bus. As soon as the temperature sensors placed in the smart home are decided to give values related to the presence of fire, based on fuzzy rules followed, the alarm will be activated, and this information can be provided to the user in an interface. Moreover, Molina-Pico et al. [129] have developed a fire-warning system based on a standard wireless sensor network with central and sensor nodes and meager energy consumption. Each sensor is geo-localized based on GPS, and nodes can be set up in vehicles resulting in more accessible long-range communications and specific mobility. Sensor nodes measure temperature, humidity, wind speed, and direction the same as CO and CO_2_ levels. According to the fire-warning index (FWI), atypical changes in temperature, humidity or gas detection can generate an alarm message. Communications and alarms operate perfectly during the forest fire simulations under real fire conditions.

One flame-retardant cellulose paper loaded with GO and MXene is fabricated to construct an EFWS [66]. The system is amazingly fast in response, with only 2 s of a wait when the paper reached 250 °C due to its rapid and sudden conductivity increase. After this waiting time, an alarm message is sent remotely to a display where the message of advice of “FIRE DANGER” can be read (Fig. 15a). In addition, the same message can also be visualized in the internet. Additionally, another work about MXene/GO film is prepared as an EFWS with remote Wi-Fi signal conversion, leading to a fast warning of 1 s when the temperature reaches 250 °C [116]. Fire brightness, related to the sample’s temperature increase and its conductivity, can be remotely monitored online, as message alarms of advice are sent wirelessly using LoRa protocol and could be visualized via the internet. Furthermore, in Ref. [96], thermoelectric units could be connected in series on one paper chip, leading to remarkable voltage signals in the presence of a temperature difference of 35 K (Fig. 15b). An alarm is activated when this temperature difference is reached at the early stage of any fire circumstance.

According to the above three crucial aspects, as well as the multifunction of current EFWSs, a comprehensive summary is provided in Table 2 for a profound understanding of the achievements of EFWS.

**Table 2:** Summary of the state of the art of EFWSs in terms of sensitivity, signal conversion, and multifunction

EFWSs		Sensitivity				Signal Conversion Type		Power Supply		Multi-functions	Refs
Types	Working mechanism	Response time calculation way	Response temperature	Response time	Durability	Tradition	Wireless	External	Self-power		
MF@GOWR	Thermal reduction of GO	Trigger electrical resistance	300 °C	33 s	–	Yes	–	Yes, ~ 29 V	–	Hydrophobicity, reversible compressibility	[18]
GO/Silicone coatings	Thermal reduction of GO	Light-on State	–	2 s—3 s	38 s	Yes	–	Yes, ~ 24 V	–	hydrophobicity	[64]
FGO/CNT@WPP	Thermal reduction of GO	Trigger electrical resistance	500 °C	7 s	–	Yes	–	Yes, ~ 40 V	–	–	[81]
MXene/GO@ Cellulose paper	Thermal reduction of GO	Electrical resistance	250 °C	2 s	–	Yes	Yes	Yes	–	–	[66]
MXene@GO-PTS film	Thermal reduction of GO	Electrical resistance	250 °C	1 s	–	Yes	Yes	Yes	–	–	[116]
Go/FC nanocoatings	Thermal reduction of GO	Light-on state	–	2.4 s	–	Yes	–	Yes	–	Self-healing	[62]
SPI/MSF-g-COOH/CA/GN film	Carbonized carbon layer structure	Light-on state	–	1 s	15 s	Yes	–	Yes	–	Flexibility, hydrophobicity	[82]
PGO@HN/GF paper	Thermal reduction of GO	Light-on state	126.9 °C	2 s	5 min	Yes	–	Yes	–	Flexibility	[80]
Silane-GO paper	Thermal reduction of GO	Light-on state	–	1.6 s	–	Yes	–	Yes, ~ 6 V	–	Flexibility, acidic/alkaline tolerance	[131]
BP-MoS_2_/GO film	Thermal reduction of GO	Light-on state	–	~ 1 s	–	Yes	–	Yes	–	–	[61]
APP/GO/silane coatings	Thermal reduction of GO	Electrical resistance	300 ℃	11.2 s	–	Yes	–	Yes	–	Hydrophobicity	[84]
AgNW@Fe_3_O_4_	Semiconductor properties	Electrical resistance	< 100 ºC	2 s	15 min	Yes	–	Yes	–	Repeatability	[38]
MMT/chitosan/A-CNT aerogel	Semiconductor properties	Light-on state	–	0.25 s	40 s	Yes	–	Yes	–	–	[42]
CB@KF-PVA-CNT	Semiconductor properties	-	350 ℃	8 s	–	Yes	–	Yes	–	–	[43]
MXene,/PEG film MXene/PVP film	Semiconductor properties	Electrical resistance	Flame	1.8 s / 1 s	–	Yes	–	Yes, ∼36 V	–	Reusability, hydrophobicity, weather-resistant	[91]
PMS	Color change	Color change	275 °C	20 s	–	–	–	–	–	–	[57]
POSS-Metal films	Color change	Color change	150 °C	–	–	–	–	–	–	Repeatability	[93]
MXene, SMPU	Shape memory	Shape change	Tg of SMPU	11 s	–	Yes	–	Yes, 5 V	–	Self-cutting, reusability	[92]
PCL, Ag layer	Shape memory	Shape change	45 ºC	5 s		Yes	Yes	Yes	–	Self-cutting	[95]
PEDOT:PSS/WPU/CNT	Thermoelectric effect	Voltage change	–	∼ 0.7 s	–	–	–	Yes	–	Reusability, hydrophobicity	[44]
PI@MXene aerogel	Thermoelectric effect	Voltage change	225 ℃	5 s	–	Yes	–	Yes	–	Reusability	[35]
Ionic liquids [EMIm][TFSI] and [EMIm][Ac]	Thermoelectric effect	Voltage change	–	–	–	Yes	–	Yes	–	Reusability	[96]
MXene, CCS	Thermoelectric effect	Voltage change	–	3.8 s	–	Yes	–	Yes	–	Reusability	[39]
Ag_2_Se/AgNW/PVB and CCS/MMT	Thermoelectric effect	Voltage change	7.4 s at 100 °C 2.7 s at 200 °C	2 s	60 s	Yes	–	–	Yes	Reusability, self-healing	[55]
MMT/UPC and MXene	Thermoelectric effect	Voltage change	–	3.1 s	–	Yes	–	–	Yes	Self-healing, reusability	[67]
CA/Fe_3_O_4_ NPs, and AgNWs	Thermoelectric/Triboelectric	Voltage change	–	2 s	–	Yes	Yes	Yes	Yes	Self-healing, reusability, hydrophobicity	[54]

*MF* melamine formaldehyde sponge; *GOWR* graphene oxide wide-ribbon; *FGO* functionalized graphene oxide; *CNTs* carbon nanotubes; *WPP* wood pulp paper; *MXene* Ti_3_C_2_; *PTS* phenyltrimethoxysilane; *FC* Functional cellulose; *SPI* soy protein isolate; *MSF-g-COOH* sisal cellulose microcrystals; *CA* Citric acid; *GN* graphene nanosheets; *PGO* polydopamine-modified GO; *HN* hydroxyapatite nanowires; *GF* glass fiber; *BP* black phosphorene; *MoS*_*2*_ molybdenum disulfide; *APP* hybrid ammonium polyphosphate; *AgNW* silver nanowires; *MMT* montmorillonite; *CB* carbon black; *PVA* polyvinyl alcohol; *PEG* polyethylene glycol; *PVP* polyvinyl pyrrolidone; *PMS* phthalocyanines precursor molecular sensor; *POSS* polyhedral oligomeric silsesquioxane; *SMPU* shape memory polyurethane; *PCL* poly(ε-caprolactone); *PEDOT:PSS* poly(3,4-ethylenedioxythiophene) polystyrenesulfonate; *WPU* waterborne polyurethane; *PI* polyimide; [EMIm][Ac]:1-ethyl-3-methyl-imidazolium acetate; [EMIm][TFSI]: 1-ethyl-3-methylimidazolium bis(trifluoromethylsulfony)imide; *CCS* carboxymethyl chitosan; *PVB* polyvinyl butyral; *UPC* 2-ureido-4[1H]-pyrimidinone-containing cellulose; *CA* calcium alginate; *NPs* nanoparticles

## Challenge and Prospect

While substantial advances have been reached in fire safety by fire-warning detection, there is still an excellent path for improvement in future, motivated by the significance of this topic. Some limitations and challenges appear in various aspects, especially the sensitivity and warning signal conversion of fire-warning systems, etc.

### Response Temperature

The advantage of warning during the early combustion process is the critical point that distinguishes between the fire-control management of fire-warning systems and other fire-control managements. The response temperature at which the alert can occur is the focus of fire control, which is a key factor in the judgment of sensitivity for fire-warning systems. Theoretically, the lower response temperature is more conducive to implementing timely fire management. However, most of the reported response temperatures are over 300 °C. Still, few fire-warning systems presented a low-temperature response. For example, in terms of GO-based fire-warning systems, most of these researches achieved a fast response. However, it is difficult to obtain a fast but low-temperature response due to the restricted minimum thermal reduction temperature of GO. Therefore, how to accomplish a lower response temperature but maintain sensitivity will be critical for the development of fire-warning systems.

### Response Time

#### Inconsistency in response time

Although early fire-warning systems have developed rapidly in recent years, there are still no uniform parameter standards for relevant tests, such as response time to the sensitivity. The sensitivity is the critical factor for early fire-warning systems, which basically consists of response temperatures, response time, and working stability. However, up to now, there are no standards on how to identify the response time. For example, when the trigger electrical resistance is selected for response time calculation, one hypothesis is the set trigger electrical resistance in various systems is different, which affects the direct contrast of response time between fire-warning systems. Moreover, trigger electrical resistance may also be impacted by the electrical circuit, such as voltage and lamp power. In fact, response time calculated by the light-on state has a similar drawback. Therefore, it does not wise to assess the warning properties of fire-warning systems only based on the response time. In this context, the establishment of a feasible and universal model for assessing the response time of fire-warning systems will be expected.

#### Durability of response time

Persistence of warning is another vital property for a fire-warning system. The long-time warning is helpful to enhance the warning signal, in particular to the fire-warning system with the traditional signal conversion method. However, in the state of the art, the durability of response time for a fire-warning system is rarely mentioned in the reported fire-warning systems, mainly due to the low stabilization of fire-warning systems in high temperature or fire conditions. Therefore, how to improve the durability of response time is still a challenge for the current fire-warning systems.

### Up-Scaling of Fire-Warning Systems

Up to the present, a study on early fire-warning systems was mainly implemented at the laboratory level via bench-scale fire scenarios. Up-scaling of fire-warning systems and studying them in large fire conditions are still very challenging. Definitely, in order to advance such studies, a series of parameters, such as fire intensity, stabilization of the fire-warning system, and size effects, have to be taken into account in future. In the end, a correlative relationship in fire-warning systems between bench-scale and up-scale is expected to be established.

## Perspectives

### Deeply Understanding of Working Mechanism

Although part of the working mechanisms of fire-warning systems has been studied, deep investigations on the mechanisms are still needed since most studies currently only focus on the performance of fire-warning systems, such as sensitive response time. For example, the presence of NaCl to modified RGO gave a rapid flame response through a self-cutting performance [132]. In case of fully understanding its working mechanism, it will be constructive to design new generation fire-warning systems. Moreover, the current studies on the mechanism were mainly related to the GO-based fire-warning systems, while few investigations on the mechanisms are to other fire-warning systems, such as semiconductor-based fire alarms, and thermochromic material-based fire alarms, thermoelectric-based fire alarms, shape change-based smart EFWS, and triboelectric nanogenerator-based fire alarms. In fact, the sensitivity of fire alarms may be influenced by the humidity and extreme external conditions as well. Further profound studies on these fire alarm systems will be needed.

### Combination with Artificial Intelligence and Simulation

Integration with artificial intelligence is an approach gaining attention in any scientific field. By means of predictive machine learning algorithms, it will be possible to achieve more comprehensive and efficient fire detection. As the first step to prevent fire hazards is the detection step of possible fire outbreaks and sensitive areas, the first studies using machine learning have been capable of developing different algorithms for mapping forest fire susceptibility, fire spread, and mapping of burned areas and also for possible building fires. Up to now, there is no specific study that has been aimed at integrating artificial intelligence into smart fire-warning systems. Nevertheless, with the information provided by more and more fundamental parameters concerning fire-warning systems, such as response time based on trigger electrical resistance, a simulated model could be established to predict suitable candidate materials for developing smarter future fire-warning systems.

### Multifunction of Fire-Warning Systems

So far, most of the reported fire-warning systems only focus on flame retardancy and early warning performance, while other properties of materials are not well considered. The development of comprehensive performance for early fire-warning systems may further extend their applications. In our opinion, non-toxic and environmentally friendly materials gradually become an inevitable choice in the development of the early fire-warning system, which can further promote the development of ecological environmental protection materials. In addition, because of the different application scenarios caused by the variability of the environment, it is necessary to fully consider the effectiveness of the early fire-warning systems in harsh environments, such as windy weather, foggy weather, and rainy weather. Correspondingly, it involves the hydrophobicity, flexibility, fire resistance, etc., of the early fire-warning systems. Taking into account, these properties can further improve the feasibility of fire-warning systems in harsh environments.

But new technologies are being implemented in conjunction with early fire-warning systems, to develop the comprehensive performances with the exception of fire-warning properties. This can actively accelerate the cooperation with other research, such as thermoelectric, nano-triboelectric generators, and wind-induced electricity. Moreover, the design and analysis of advanced materials for EFWSs should be vigorously promoted to accomplish their multifunction. This multifunction consists of self-healing, reusability, humidity detection, water resistance, and gas selection performances, which is an inevitable development trend to broaden the practical application scenarios of early fire-warning systems. In addition, integration of fire sensors with e-textile offers a quite promising for the designability of early fire-warning systems, which can open the idea of protecting firefighters and localization. Nonetheless, other fabrics, such as cotton, and flax, have been mixed with thermosensitive materials with clear potential for smart clothing and e-textile. Similarly, self-sustaining fire alarms could provide a remote control of inhabited areas and factories in the outer suburbs.
