# Correction to: Recent Advances on Early-Stage Fire-Warning Systems: Mechanism, Performance, and Perspective

**DOI:** 10.1007/s40820-022-00973-8

**Published:** 2022-11-23

**Authors:** Xiaolu Li, Antonio Vázquez-López, José Sánchez del Río Sáez, De-Yi Wang

**Affiliations:** 1 IMDEA Materials Institute, C/Eric Kandel, 2, 28906 Getafe, Madrid, Spain; 2 E.T.S. de Ingenieros de Caminos, Universidad Politécnica de Madrid, Calle Profesor Aranguren 3, 28040 Madrid, Spain; 3 E.T.S. of Design and Industrial Engineering (E.T.S.I.D.I), Department of Electrical, Electronics, Automation Engineering and Applied Physics, Universidad Politécnica de Madrid (UPM), C/Ronda de Valencia 3, 28012 Madrid, Spain

**Correction to: Nano-Micro Lett. (2022) 14:197** 10.1007/s40820-022-00938-x

In the original publication, the name of third author has been published incorrectly as “Sánchez del Río Saeza, J” and this has corrected as “Sánchez del Río Sáez, J”.

The original article has been updated.

